# Signaling Through SCFA Receptors Gpr43 and Gpr109a Drives Pro‐Inflammatory M1 Macrophage Polarization in Periodontitis

**DOI:** 10.1155/mi/3542645

**Published:** 2026-04-08

**Authors:** Kun Liu, Ruolin Wang, Lingxue Kong, Lisi Ai

**Affiliations:** ^1^ Department of Oral and Maxillofacial Surgery, Jinan Stomatological Hospital, Jinan, 250001, China, jnskqyy.com; ^2^ Department of Central Laboratory, Jinan Stomatological Hospital, No. 101 Jingliu Road, Shizhong District, Jinan, 250001, Shandong, China, jnskqyy.com; ^3^ Jinan Key Laboratory of Oral Diseases and Tissue Regeneration, Shandong Provincial Key Medical and Health Laboratory of Oral Diseases and Tissue Regeneration, Jinan, 250001, China; ^4^ Department of Periodontics and Oral Mucosa, Jinan Stomatological Hospital, Jinan, 250001, China, jnskqyy.com

**Keywords:** macrophage, metabolic reprograming, microbiota metabolites, multiomics analysis, periodontitis, single-cell RNA sequencing

## Abstract

**Background:**

This study investigated the link between an imbalance in microbiota‐derived short‐chain fatty acids (SCFAs) and dysregulated host immunity in periodontitis, focusing on the metabolic reprograming of inflammatory macrophages.

**Methods:**

We conducted a systematic review and meta‐analysis of SCFA levels. Bulk RNA sequencing (RNA‐seq) data from human gingival tissue (*n* = 24) were analyzed for differential expression and pathway enrichment, with immune cell composition estimated by Cell‐type Identification by Estimating Relative Subsets of RNA Transcripts (CIBERSORT). Mouse single‐cell RNA‐seq (scRNA‐seq) data were integrated, and myeloid cells (MCs) were subset for detailed clustering, differential analysis, and pseudotime trajectory reconstruction.

**Results:**

Meta‐analysis indicated a decreasing, though nonsignificant, trend in butyrate levels in periodontitis. Bulk RNA‐seq identified 692 differentially expressed genes (DEGs) enriched in immune and cytokine signaling pathways. Immune deconvolution revealed an increased proportion of M1 macrophages and Tregs, alongside a decrease in M2 macrophages. Single‐cell analysis confirmed the significant expansion of M1‐like macrophages, which highly expressed SCFA receptors (Gpr43 and Gpr109a), inflammatory transcription factors (Nfkb1 and Hif1a), and effector molecules (Il1b). The pseudotime trajectory demonstrated a continuous M2‐to‐M1 polarization, marked by a decline in M2 markers and a rise in M1 markers.

**Conclusion:**

Periodontitis is characterized by SCFA metabolic imbalance and a shift in MCs toward a pro‐inflammatory M1 state. The upregulation of SCFA receptors and the NF‐κB/HIF‐1 axis in M1 macrophages suggests an “SCFA receptor–metabolic sensing–inflammatory transcription” mechanism drives disease progression, providing a rationale for therapeutic strategies targeting this pathway.

## 1. Introduction

Periodontitis is a chronic destructive disease characterized by the colonization of pathogenic bacteria at the gingival margin and periodontal pockets, triggering a host immune‐inflammatory response. Its primary manifestations include gingival inflammation, alveolar bone resorption, and loss of periodontal support tissues [1]. According to global burden of disease studies, the prevalence of severe periodontitis is ~10%–15%, making it the leading cause of tooth loss in adults and significantly affecting chewing, speech, and quality of life [2–4]. A substantial body of research indicates an association between periodontitis and systemic diseases such as cardiovascular disease, diabetes, rheumatoid arthritis, chronic kidney disease, and certain cancers. This correlation may be attributed to prolonged inflammation and the presence of inflammatory mediators in the bloodstream [5]. The progression of periodontitis results from host–microbe interactions; while immune responses can eliminate pathogens and maintain homeostasis, they may also lead to tissue destruction if imbalanced [6, 7]. Although conventional debridement and anti‐infective therapies can alleviate symptoms, the critical molecular mechanisms driving persistent inflammation and tissue damage remain inadequately elucidated. In particular, the interaction mechanisms between immune regulation and metabolic signaling warrant further investigation [1].

The oral cavity hosts ~700 distinct bacterial species, forming a complex microbial community. The metabolic products of these bacteria play a crucial regulatory role in host–microbe interactions [8, 9]. Short‐chain fatty acids (SCFAs) are the primary metabolic byproducts of oral microbiota fermentation of polysaccharides, proteins, and peptides, including acetate, propionate, butyrate, and isovalerate [10, 11]. SCFA not only provides energy to colonic epithelium but also modulates immune cell transcription programs and functions by binding to G‐protein‐coupled receptors (such as G protein‐coupled receptor 43 (Gpr43), Gpr41, G protein‐coupled receptor 109A [Gpr109a]), or inhibiting histone deacetylases [12, 13]. In systemic immune regulation, SCFAs promote regulatory T cells (Treg) differentiation, suppress pro‐inflammatory cytokine release, and induce anti‐inflammatory macrophage phenotypes [10, 14]. However, in periodontitis, significant alterations in microbiome composition and metabolic profiles may lead to fluctuations in local SCFA levels, disrupting immune homeostasis and triggering aberrant inflammatory responses [15, 16]. Previous studies suggest that SCFA levels in saliva, subgingival plaque, and gingival crevicular fluid (GCF) of periodontitis patients may decrease; however, variations in sample sources, detection techniques, and populations among studies result in heterogeneity. Notably, the direction and magnitude of changes in individual SCFA components differ [17, 18]. Therefore, a systematic meta‐analysis is necessary to integrate existing evidence to elucidate the trends of SCFA changes in periodontitis and their immunological significance [19].

Macrophages are pivotal innate immune cells within the inflammatory microenvironment of periodontitis, playing critical roles in pathogen recognition, inflammation amplification, and tissue repair. They exhibit high plasticity and can differentiate into pro‐inflammatory M1 and anti‐inflammatory/repair‐oriented M2 phenotypes [20]. The M1 phenotype is induced by IFN‐γ and LPS [21, 22], characterized by high expression of pro‐inflammatory cytokines such as TNF‐α, IL‐1β, IL‐6, and reactive oxygen species, which contribute to tissue destruction. Conversely, the M2 phenotype is stimulated by IL‐4 and IL‐13, secreting anti‐inflammatory cytokines like IL‐10 that promote repair and angiogenesis [23]. Immunometabolism studies have revealed a close association between macrophage polarization and their metabolic pathways: M1 macrophages rely on aerobic glycolysis, while M2 macrophages depend on oxidative phosphorylation and fatty acid oxidation [23, 24]. SCFAs can modulate macrophage metabolism and polarization direction through receptor signaling or epigenetic mechanisms; activation of Gpr43 and Gpr109a can enhance the anti‐inflammatory phenotype while suppressing inflammatory genes [25, 26]. However, in chronic inflammation such as periodontitis, macrophages may undergo a phenotypic shift from M2 to M1, accompanied by the activation of nuclear factor kappa‐light‐chain‐enhancer of activated B cells (NF‐κB) and hypoxia‐inducible factor 1 (HIF‐1), forming a pro‐inflammatory positive feedback loop that drives persistent inflammation [27, 28]. This inflammatory metabolic reprograming could be a critical factor in the chronicity and recalcitrance of periodontitis.

The advancement of multiomics technologies has provided novel tools for elucidating the mechanisms of complex diseases [29]. Meta‐analysis, which integrates multiple studies at the population level, offers more robust effect estimates and is suitable for assessing trends in SCFA changes [30, 31]. RNA sequencing (RNA‐seq) can reveal differentially expressed genes (DEGs) and enriched pathways, reflecting molecular networks within inflammatory microenvironments. Algorithms such as Cell‐type Identification by Estimating Relative Subsets of RNA Transcripts (CIBERSORT) can infer changes in immune cell proportions [32, 33], linking molecular alterations to cellular composition. Single‐cell RNA‐seq (scRNA‐seq) allows for the analysis of heterogeneity among different cell types and subpopulations, and when combined with pseudo‐time analysis, it can reconstruct trajectories of cellular state transitions. This approach captures the dynamic process and key regulatory factors involved in macrophage polarization from M2 to M1 [26, 34]. Such an integrative strategy across hierarchical levels and data types provides a comprehensive depiction of the “microbiome metabolism—immune cell composition—metabolic reprograming” landscape in periodontitis, laying the groundwork for identifying new therapeutic targets [35, 36]. However, there is currently a paucity of studies integrating SCFA metabolic profiles, immune cell composition, and scRNA‐seq‐derived macrophage polarization trajectories in periodontitis research, limiting a systematic understanding of its mechanisms [37, 38].

This study aims to systematically investigate the relationship between SCFA metabolic dysregulation and host immune response abnormalities in periodontitis using an integrative multiomics approach. By analyzing the role of SCFAs in immune cell polarization, we aim to uncover their potential mechanisms in the chronic progression of periodontitis and further explore how immune metabolic reprograming drives the persistence of inflammation and the progression of tissue damage. This study will provide deeper insights into the impact of SCFA metabolic imbalances on immune cell function, inflammatory responses, and tissue repair, elucidating the complex interactions between metabolism and immunity in periodontitis. This research will not only enhance our understanding of the pathogenesis of periodontitis and advance the theoretical framework of immune metabolism but also offer novel therapeutic targets and intervention strategies for clinical treatment. For instance, modulating SCFA levels or targeting their receptor signaling pathways may provide a solid molecular basis for controlling inflammation and promoting tissue repair in periodontitis, thus enabling more precise and personalized treatment approaches.

## 2. Materials and Methods

### 2.1. Literature Search Strategy

To systematically evaluate the expression changes in microbial metabolic products in patients with periodontitis, a literature search was conducted using three databases: PubMed, Embase, and Web of Science. The search was confined to publications from January 1, 2000 to December 31, 2024. The search strategy employed a combination of controlled vocabulary and free‐text terms as follows: ((“Periodontitis”[Mesh]) OR (Pericementitis[Title/Abstract]) OR (Periodontitides[Title/Abstract])) AND ((“Fatty Acids, Volatile”[Mesh]) OR (SCFA[Title/Abstract]) OR (butyrate[Title/Abstract]) OR (propionate[Title/Abstract]) OR (acetate[Title/Abstract])). The studies were limited to those involving human subjects (Humans[Mesh]). All identified literature was imported into EndNote X9 (Clarivate Analytics, USA) for deduplication.

### 2.2. Criteria for Literature Screening and Inclusion/Exclusion

The initial and subsequent screenings of the literature were conducted independently by two researchers. The initial screening was based on titles and abstracts, while the subsequent screening involved a full‐text assessment. The inclusion criteria were as follows: (1) studies involving human periodontitis patients and healthy controls; (2) application of high‐throughput metabolomics methods such as gas chromatography–mass spectrometry (GC–MS) or LC–MS for detecting microbial metabolites; (3) quantitative expression of SCFA metabolites reported; (4) provision of extractable mean ± standard deviation or median + interquartile range data; (5) use of a case‐control study design. The exclusion criteria included: (1) animal experiments or in vitro studies; (2) absence of specific quantitative information on SCFAs or other microbial metabolites; (3) abstracts, reviews, conference papers, or duplicate publications; (4) studies containing only a single group without a control group.

### 2.3. Assessment of Literature Quality

All studies included in this analysis were observational clinical studies with a case–control design and did not involve random allocation or interventional procedures. All included studies were evaluated for risk of bias using the Newcastle–Ottawa Scale (NOS) tool. This instrument comprises three components: Selection (four points), Comparability (two points), and Outcome Assessment or Exposure Measurement (three points), with a maximum score of nine points. The scoring was conducted using Microsoft Excel 2016 (Microsoft Corp., USA). Studies achieving a score of ≥7 were classified as high‐quality, those scoring between 5 and 6 as medium‐quality, and scores below 5 as low‐quality. Given that all included studies were observational in nature, risk‐of‐bias assessment tools specifically designed for randomized controlled trials were not applied.

### 2.4. Data Extraction and Standardization

Extract data from each study, including author, publication year, country, sample size, mean age, metabolite detection method, type of sample analyzed (e.g., saliva, subgingival plaque, and feces), specific metabolite names, and their expression levels (mean ± standard deviation or median + interquartile range). For studies reporting data as median and interquartile range, use the method by Wan et al. [39] to estimate the mean and standard deviation under a normal distribution. All data are organized in an Excel file and imported into the R software for subsequent meta‐analysis.

### 2.5. Meta‐Analysis and Heterogeneity Assessment

The meta‐analysis was conducted in R (version 4.2.2) utilizing the “meta” package (version 6.5‐0) and the “metafor” package (version 4.2‐0). The Standardized Mean Difference (SMD) and its 95% confidence interval (CI) were calculated. Heterogeneity was evaluated using the *I* ^2^ statistic: an *I* ^2^ > 50% indicates moderate‐to‐high heterogeneity, prompting the use of a random‐effects model (DerSimonian–Laird method); otherwise, a fixed‐effects model was employed. A *p*‐value < 0.05 was considered statistically significant. Considering the limited number of included studies and the potential heterogeneity among them, the results of the meta‐analysis were primarily used to explore trend associations rather than to support causal inferences.

### 2.6. Subgroup and Sensitivity Analyses

To investigate potential sources of heterogeneity, subgroup analyses were conducted based on sample type (saliva, GCF, and gingival tissue), detection platform (GC–MS, LC–MS), and geographical region (Asia, Europe, and America). The “byvar” parameter in the meta package was used to specify grouping variables. Sensitivity analysis was performed using the leave‐one‐out method to observe changes in the pooled effect size and heterogeneity, thereby assessing the robustness of the analysis. Given that some subgroups included a limited number of studies, the corresponding results were used solely to describe potential sources of heterogeneity and were not interpreted as independent conclusions.

### 2.7. Effect Size Ranking, Meta‐Regression, and Robustness Assessment

To more systematically compare the magnitude of changes in different SCFAs in periodontitis, the pooled effect sizes of each SCFA were further ranked without relying solely on statistical significance. The absolute SMD (|SMD|) was used as an indicator of effect size magnitude, and the relative ranking was presented in a visualized manner. To explore sources of between‐study heterogeneity, a multivariable random‐effects meta‐regression model (rma.mv) implemented in the *metafor* package was applied. Detection method (high‐performance capillary electrophoresis [HPCE] or high‐performance liquid chromatography [HPLC]), sample source (GCF or saliva), and study population/country were included as covariates. Model fitting was performed using the restricted maximum likelihood (REML) approach. In addition, a leave‐one‐out analysis was conducted to assess the influence of individual studies on the pooled estimates and to evaluate the robustness of the results. To address the issue of multiple comparisons, false discovery rate (FDR) correction was considered in the interpretation of results, with greater emphasis placed on the structure and consistency of effect sizes rather than on isolated statistical significance.

### 2.8. Analysis of Publication Bias

The funnel() function from the “metafor” package in R was employed to construct funnel plots for the assessment of publication bias. Egger’s linear regression test was utilized to quantify bias, with a significance level set at *p* < 0.1. In cases where significant bias was detected, the Trim‐and‐Fill method was subsequently applied for correction, and the corrected SMD values were reported.

### 2.9. Extraction and Synthesis of Correlation Information Between SCFAs and Inflammatory Markers

To further explore whether changes in SCFAs in periodontitis are associated with inflammatory status and to assess their potential indirect links to pathogen‐driven processes, we systematically extracted and summarized available correlation information between SCFAs and inflammatory markers from the studies included in the existing meta‐analysis framework. Specifically, after full‐text review of each eligible study, we identified and extracted author‐reported correlations between SCFAs (including acetate, propionate, butyrate, isovalerate, and among others) and local inflammatory indicators. Inflammation‐related outcomes preferentially included inflammatory mediators measured in GCF or saliva (such as IL‐1β, TNF‐α, IL‐6, IL‐8, and MMP‐8). If molecular inflammatory markers were not reported, correlations with clinical inflammatory phenotypes (e.g., bleeding index, probing depth, or clinical attachment loss) were additionally recorded. For each SCFA–inflammatory marker pair, the type of correlation coefficient (Pearson or Spearman), the correlation coefficient value, the corresponding *p*‐value, and sample size were extracted. If a study did not report correlation analyses, this was noted as “NR” (not reported) in the summary table.

Given the limited number of included studies and the substantial heterogeneity across studies in terms of measured outcomes, sample types, and statistical approaches, no imputation or reconstruction of missing correlation coefficients was performed. When the same SCFA–inflammatory marker association was explicitly reported in two or more studies, Fisher’s *z* transformation was applied to standardize correlation coefficients, followed by exploratory pooling using a random‐effects model. Correlation results reported in only a single study were summarized descriptively. All correlation findings are presented in supporting tables and are intended to support exploratory interpretation of the potential relationships between SCFA alterations and inflammatory status in periodontitis, rather than to serve as evidence for causal inference.

### 2.10. Retrieval and Screening of Public Transcriptomic Datasets

To minimize selection bias and ensure the completeness of data sources, a systematic search and screening of human gingival tissue transcriptomic datasets related to periodontitis were conducted in the Gene Expression Omnibus (GEO) database. Search terms were constructed around disease and tissue origin, including “periodontitis/periodontal disease” and “gingival tissue/gingiva,” with filters applied for *Homo sapiens* and high‐throughput RNA‐seq‐based expression profiling (RNA‐seq). Initial screening was performed based on dataset titles, abstracts, and sample annotation information, retaining datasets that included both periodontitis and healthy control samples, used gingival tissue as the sample type, and had clearly defined group information. Secondary screening further assessed the availability of raw data, confirmation that samples were obtained at untreated baseline status, platform consistency, and the absence of major confounding factors (such as postdrug or postsurgical sampling, nongingival or mixed tissue sources). Datasets that did not meet these criteria were excluded, and candidate datasets along with detailed reasons for exclusion were compiled into a screening list. The GEO dataset search and screening workflow, as well as exclusion rationales, are summarized in Supporting Information 1: Table S1.

### 2.11. Public Data Download

Based on the study objectives and data availability, GSE173082 was ultimately selected as the data source for batch RNA‐seq analysis. This dataset comprises high‐throughput sequencing‐based transcriptomic profiles of human gingival tissues, includes both periodontitis and healthy control samples with clearly annotated group information, and is well suited for downstream analyses such as differential expression, pathway enrichment, and immune infiltration inference. In addition, its study design incorporates multiomics information related to periodontal health and disease, facilitating transcriptional characterization of inflammatory and immune processes and integration with subsequent mechanistic analyses. The final cohort included samples from patients with periodontitis (*n* = 12) and healthy controls (*n* = 12). Raw sequencing data were downloaded using the SRA Toolkit and converted to FASTQ format, after which quality control, alignment, and read counting were performed following the same pipeline described above.

### 2.12. Alignment and Gene Counting

The cleaned FASTQ sequences were aligned to the human genome reference sequence GRCh38 (downloaded from GENCODE, Release 41) using HISAT2 (v2.2.1). The alignment was performed with the parameter—rna‐strandness RF, while other parameters were set to default values. The resulting SAM files were converted to binary BAM format and sorted using SAMtools (v1.13). Subsequently, featureCounts (v2.0.1, Subread package) was employed to count the number of reads for each gene based on the annotation file provided by GENCODE (GTF format), with parameters set as ‐t exon ‐g gene_id, specifying counting at the transcript exon level.

### 2.13. Differential Expression Analysis

The gene count matrix was imported into the Differential Expression Analysis for Sequence Count Data 2 (DESeq2) package (v1.38.3, Bioconductor) for normalization and differential expression analysis. Samples were grouped into “periodontitis” and “healthy control” categories, with dispersion estimation and statistical testing conducted using the DESeq function. DEGs were identified based on criteria of |log_2_FoldChange| > 1 and FDR < 0.05. A total of 692 DEGs were identified, with 574 upregulated and 118 downregulated. The volcano plot was generated using the “EnhancedVolcano” package (v1.16.0), while a heatmap illustrating the top 50 genes with significant expression changes was created using the “pheatmap” package (v1.0.12).

### 2.14. Principal Component Analysis (PCA)

PCA was conducted on the normalized expression matrix using the prcomp function, with visualization achieved through the “ggplot2” package (v3.4.2). Principal components PC1 and PC2 accounted for 34.7% and 22.1% of the variance, respectively, indicating a distinct separation in overall transcriptional expression patterns between the two sample groups.

### 2.15. Gene Ontology (GO) and Kyoto Encyclopedia of Genes and Genomes (KEGG) Functional Enrichment Analysis

DEGs were subjected to GO and KEGG pathway enrichment analysis using the ClusterProfiler package (v4.6.2). The GO analysis encompassed three categories of annotations: biological process (BP), molecular function (MF), and cellular component (CC). For the KEGG analysis, the species parameter was set to “hsa,” and the enrichment *p*‐Value was adjusted using the Benjamini–Hochberg correction method. Pathways with an FDR less than 0.05 were considered significantly enriched. Visualization of significant pathways and their enrichment scores was performed using the “ggplot2” and “enrichplot” packages (v1.18.4).

### 2.16. Estimation of Immune Cell Infiltration Using CIBERSORT

The immune cell composition was estimated using the CIBERSORTx online platform, applied to the RNA‐seq expression matrix. A normalized TPM expression matrix was uploaded, utilizing the LM22 signature matrix as a reference, with the permutation number set to 1000 and quantile normalization disabled. The output provides the relative proportions of 22 types of immune cells within each sample. Statistical analysis involved comparing the differences in immune cell proportions between two groups using the Wilcoxon rank‐sum test, and visual representations were generated using box plots from the “ggplot2” package. It should be noted that bulk RNA‐seq reflects overall transcriptional profiles at the tissue level, whereas single‐cell transcriptomic analysis is designed to resolve gene expression states within specific immune cell subsets. Therefore, results derived from these different analytical levels are intended to provide complementary insights rather than direct one‐to‐one gene‐level correspondence.

### 2.17. Association Analysis Between Metabolite Receptor Gene Expression and Immune Cell Proportions

To evaluate the relationship between overall expression changes of metabolite‐sensing receptor genes in bulk RNA‐seq data and immune cell composition, we further analyzed the correlations between the expression levels of GPR43 (FFAR2) and solute carrier family 5 member 8 (SLC5A8) and the proportion of M1 macrophages inferred by CIBERSORT. Spearman’s rank correlation analysis was performed using DESeq2‐normalized gene expression values to assess associations between gene expression and immune cell proportions. Correlation analyses and visualization were conducted in the R environment, with statistical significance defined as *p* < 0.05.

### 2.18. Analysis of Immune‐Related Gene Expression

Representative markers of immune cells (FOXP3, CD86, iNOS, CD163, and Arg1), inflammatory cytokines (IL1B, TNFA, and IL10), and metabolite receptors (GPR43, SLC5A8, toll‐like receptor 4 [TLR4]) were selected based on literature and the ImmPort database (https://www.immport.org/). The expression values of these genes were extracted from the DESeq2‐normalized expression matrix. Box plots were generated using the “ggpubr” package (v0.6.0) to visualize group differences, and intergroup comparisons were conducted. *p*‐Values were calculated using a two‐tailed nonparametric Wilcoxon test with a significance threshold set at *p* < 0.05.

### 2.19. Data Sources and Preprocessing

The scRNA‐seq data were sourced from the publicly available dataset GSE228635 in the GEO database. This dataset includes one healthy control mouse and three periodontitis model mice, sampled on Days 1, 4, and 7 postinduction. The raw sequencing data, downloaded in FASTQ format, was processed using Cell Ranger (v6.1.2, 10x Genomics, USA) for alignment to the reference genome (mm10) and generation of the gene expression matrix.

### 2.20. Data Standardization and Integration

Following quality control, data normalization was performed using the SCTransform method. PCA was conducted based on 3000 highly variable genes. Batch effect correction was achieved using Harmony (v0.1.0), integrating healthy controls with periodontitis samples from different time points into a unified expression matrix.

### 2.21. Dimensionality Reduction Clustering and Cell Type Annotation

Dimensionality reduction was conducted using RunPCA (the first 30 principal components), followed by t‐distributed stochastic neighbor embedding (t‐SNE) or UMAP for two‐dimensional visualization. The clustering resolution was set at 0.5. Cell type annotation was based on canonical marker genes, with cross‐validation performed using the automatic annotation results from SingleR (v2.2.0).

### 2.22. Analysis of Myeloid Cell (MC) Subpopulations

After extracting the subpopulations annotated as MCs, a sequential analysis involving ScaleData, PCA, UMAP, and clustering was conducted with a resolution set at 0.3. This process aimed to identify subtypes such as M1 inflammatory macrophages, M2 reparative macrophages, and neutrophils. Subpopulation‐specific markers were selected based on the FindAllMarkers function (Wilcoxon rank‐sum test, min.pct = 0.25, log_2_FC > 0.5).

### 2.23. Pseudotemporal Analysis

The reconstruction of MC trajectories was performed using Monocle 2 (v2.22.0). A matrix of highly variable genes was input, and dimensionality reduction was conducted via DDRTree to construct developmental trajectories of the cells. The plot_pseudotime_heatmap function was employed to analyze the dynamic expression changes of key inflammation and repair‐related genes, such as Tnf, Il1b, Nos2, Arg1, Mrc1, and Il10, along the trajectory.

## 3. Results

### 3.1. Literature Screening

**Figure 1:**
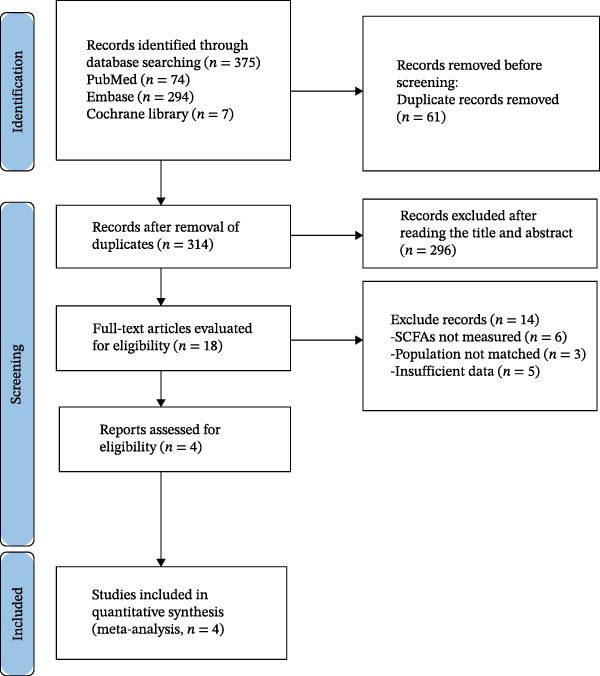
Literature screening flowchart of microbiota‐derived metabolites in patients with periodontitis. *Note:* Flowchart illustrating the literature screening process for the meta‐analysis of short‐chain fatty acid (SCFA) metabolites in periodontitis. A total of 375 records were identified through database searches (74 from PubMed, 294 from Embase, and seven from the Cochrane Library). After removal of 61 duplicates, 314 records remained for screening. Following title and abstract screening, 296 records were excluded. Eighteen articles underwent full‐text assessment, of which 14 were excluded due to absence of SCFA measurements (*n* = 6), unmatched study populations (*n* = 3), or incomplete data (*n* = 5). Ultimately, four studies were included in the quantitative synthesis (meta‐analysis).

During the systematic search phase, a total of 375 relevant literature records were obtained from three major databases, namely PubMed (74 records), Embase (294 records), and the Cochrane Library (seven records). After removing duplicates, 61 redundant records were eliminated, leaving 314 articles for initial screening based on titles and abstracts. Subsequently, 296 articles were excluded due to being irrelevant to the research theme or lacking critical information, resulting in 18 articles selected for a full‐text assessment. Under stringent inclusion criteria, 14 articles were further excluded: six did not measure SCFAs, three involved mismatched study populations, and five presented incomplete data. Ultimately, four studies were included in the meta‐analysis (Figure 1).

This study included four randomized controlled trials conducted in China and Japan. Sample sizes in these studies ranged from 12 to 40 participants, comprising both periodontitis patients and healthy controls. There was a notable age difference among the groups, with periodontitis patients being significantly older than the healthy controls, the most significant disparity occurring in the study by Hatanaka (66.85 ± 9.55 years vs. 48.5 ± 21.6 years). Regarding gender composition, most studies reported a relatively balanced male‐to‐female ratio in both the healthy and periodontitis groups. The sample types were primarily GCF or saliva, with SCFA detection techniques, including HPCE, GC–MS, and HPLC. Variations in reported SCFA types were observed across different studies, but core metabolites such as acetate (acetic acid), propionate (propionic acid), butyrate (butyric acid), isobutyrate (isobutyric acid), and isovalerate (isovaleric acid) were commonly included. Detailed basic information and quality scores of all included articles are presented in Table 1.

**Table 1:** Study characteristics.

No./author/year		① Lu/2014	② Hatanaka/2022	③ Koike/2020	④ Li /2012
Country		China	Japan	Japan	China
Study type		RCT	RCT	RCT	RCT
Total sample size (*n*)		40	30	12	37
Group					
Age (years, mean ± SD)	Healthy controls	26.2 ± 3.0	48.5 ± 21.6	32.6 ± 6.1	28.9 ± 7.4
	Periodontitis patients	4.5 ± 4.1	66.85 ± 9.55	53.1 ± 13.7	50.0 ± 5.4
Gender (Male/Female)	Healthy controls	8/12	4/6	—	14/7
	Periodontitis patients	9/11	4/16	—	7/9
Sample source		GCF	Saliva	Saliva	GCF
Analytical platform		HPCE	GC–MS	HPLC	HPCE
Key SCFAs reported		abce	a	abcd	bce

*Note:* a = acetic acid; b = propionic acid; c = butyric acid; d = isobutyric acid; e = isovaleric acid.

Abbreviations: GCF, gingival crevicular fluid; GC–MS, gas chromatography–mass spectrometry; HPCE, high performance capillary electrophoresis; HPLC, high performance liquid chromatography.

All four included randomized controlled trials were appraised using the Cochrane risk‐of‐bias assessment tool. The results indicated that most studies exhibited a low risk of bias in random sequence generation, allocation concealment, blinding of participants and personnel, blinding of outcome assessment, data completeness, and selective reporting. Specifically, Lu et al. [40] and Qiqiang et al. [41] were judged to have a low risk of bias across all evaluation domains, whereas Hatanaka et al. [42] and Koike et al. [43] presented an unclear risk in specific domains, including random sequence generation, blinding of outcome assessment, and selective reporting (Supporting Information 2: Figure S1).

Overall, all studies were assessed as low risk in the “other bias” category, with uncertainty in selective reporting and blinding of outcome assessment identified as the principal potential sources of bias.

### 3.2. Meta‐Analysis of Propionate Concentration in Patients With Periodontitis

To assess the differences in propionate levels between patients with periodontitis and healthy controls, a meta‐analysis was conducted on three studies that met the inclusion criteria. The overall combined results indicated that the difference in propionate levels between the two groups was not statistically significant (SMD = 0.06, 95% CI: −1.56 to 1.67), with high heterogeneity (*I* ^2^ = 83.0%, *τ* ^2^ = 1.7941, *p* = 0.0028; Figure 2A).

Figure 2Meta‐analysis of propionic acid expression differences in patients with periodontitis. *Note:* (A) SMD analysis of three studies comparing propionic acid concentrations between patients with periodontitis and healthy controls; (B) subgroup analysis stratified by sample type (GCF and saliva); (C) subgroup analysis stratified by analytical platform (HPLC and HPEC); (D) sensitivity analysis assessing the influence of each study on the pooled effect size; (E) funnel plot evaluating publication bias.

**Figure:**
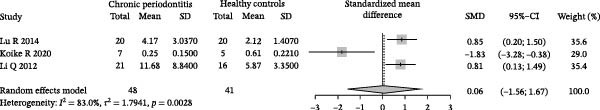
(A)

**Figure:**
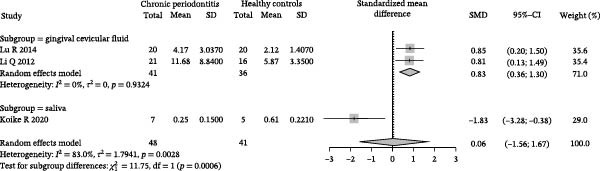
(B)

**Figure:**
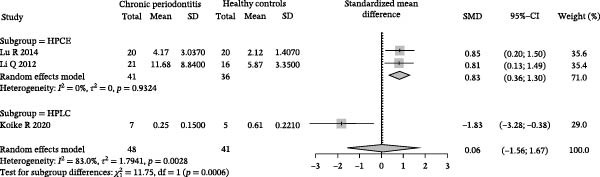
(C)

**Figure:**
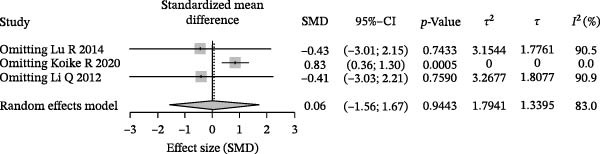
(D)

**Figure:**
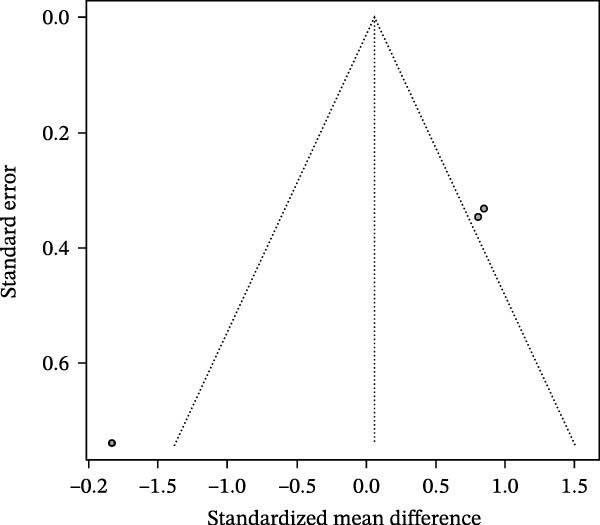
(E)

To further investigate the potential sources of heterogeneity, subgroup analyses were conducted based on sample source and analytical platform. In the sample source subgroup, the GCF group, comprising studies by Lu et al. [40] and Qiqiang et al. [41], showed a combined effect size of SMD = 0.83 (95% CI: 0.36–1.30) with significantly reduced heterogeneity (*I* ^2^ = 0%, *p* = 0.93). In contrast, the saliva group, represented by Koike et al. [43], contained only one study, showing an SMD of −1.83 (95% CI: −3.28 to −0.38), suggesting that sample source may be a critical factor affecting study consistency (Figure 2B). Correspondingly, in the analytical platform subgroup, studies using HPCE by Lu et al. [40] and Qiqiang et al. [41] demonstrated stable and consistent effects (SMD = 0.83, 95% CI: 0.36–1.30, *I* ^2^ = 0%), whereas the study using HPLC by Koike et al. [43] showed significant deviation, further emphasizing the impact of the analytical platform on quantitative results (Figure 2C).

Sensitivity analysis revealed that the overall direction of effect did not fundamentally change upon exclusion of any single study. After removing Koike et al. [43], the combined effect size increased to SMD = 0.83 (95% CI: 0.36–1.30), and heterogeneity was eliminated (*I* ^2^ = 0%), indicating that this study may be the primary contributor to heterogeneity (Figure 2D). The funnel plot distribution was largely symmetrical, and although the number of included studies was limited, no obvious risk of publication bias was observed (Figure 2E).

### 3.3. Meta‐Analysis of Butyric Acid Concentration in Patients With Periodontitis

Meta‐analysis indicated an overall decreasing trend in butyrate levels in GCF or saliva from patients with periodontitis; however, the difference did not reach statistical significance (SMD = −1.58, 95% CI: −6.53 to 3.36) and heterogeneity was substantial (*I* ^2^ = 90.9%, *p* < 0.0001; Figure 3A). Subgroup analysis by sample source revealed that butyrate levels in GCF were lower in periodontitis patients than in healthy controls (SMD = 0.77, 95% CI: −0.22 to 1.77, *I* ^2^ = 77.1%), with an even greater reduction observed in saliva samples (SMD = −7.21, 95% CI: −10.87 to −3.55; Figure 3B). Stratification by detection method showed that studies using HPCE reported a significant difference (SMD = 0.77, 95% CI: −0.22 to 1.77, *I* ^2^ = 77.1%), whereas those employing HPLC found a markedly larger negative effect size (SMD = −7.21, 95% CI: −10.87 to −3.55). Tests for subgroup differences indicated that both sample source and detection method significantly influenced the effect size (*χ* ^2^ = 17.02, df = 1, *p* < 0.0001; Figure 3C). Sensitivity analysis demonstrated that sequential exclusion of individual studies did not materially alter the pooled effect size or statistical significance, supporting the robustness of the results (Figure 3D). Funnel plot symmetry suggested a low risk of publication bias (Figure 3E).

Figure 3Meta‐analysis of butyric acid expression differences in patients with periodontitis. *Note:* (A) SMD analysis of three studies comparing butyric acid concentrations between patients with periodontitis and healthy controls; (B) subgroup analysis stratified by sample type (GCF and saliva); (C) subgroup analysis stratified by analytical platform (HPLC and HPEC); (D) sensitivity analysis assessing the influence of each study on the pooled effect size; (E) funnel plot evaluating publication bias.

**Figure:**
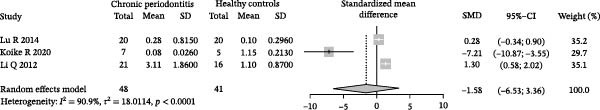
(A)

**Figure:**
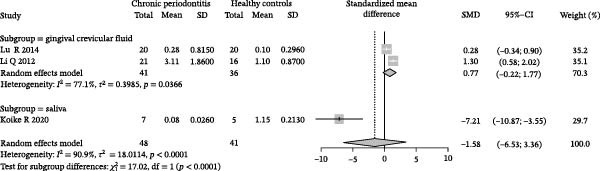
(B)

**Figure:**
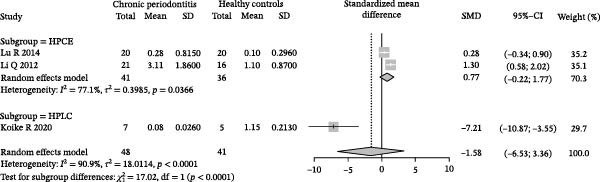
(C)

**Figure:**
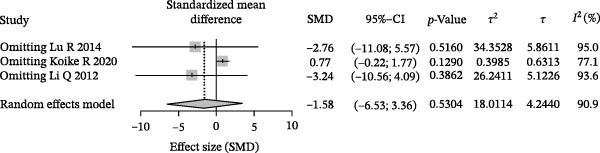
(D)

**Figure:**
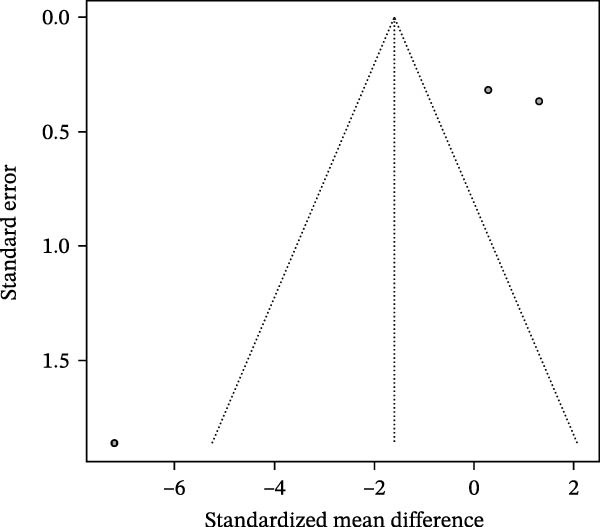
(E)

In summary, butyrate levels derived from the microbial metabolites in patients with periodontitis showed a decreasing trend in the overall analysis but did not reach statistical significance. However, a more consistent direction of change was observed in certain subgroups, suggesting that the observed variation may be influenced by sample type and detection methodology.

### 3.4. Meta‐Analysis of Acetate Concentration in Periodontitis Patients

The meta‐analysis results indicate that the overall difference in acetate levels between periodontitis patients and healthy controls does not reach statistical significance (SMD = −0.60, 95% CI: −1.41 to 0.21), with moderate heterogeneity (*I* ^2^ = 58.6%; Figure 4A). A subgroup analysis based on sample source reveals that in the GCF group, acetate levels in periodontitis patients are lower than in healthy controls (SMD = −1.02, 95% CI: −2.21 to 0.17, *I* ^2^ = 55.8%), whereas in the saliva group, the differences are more minor and inconsistent, with some studies showing negative differences (e.g., SMD = −1.80, 95% CI: −3.23 to −0.36) and others showing minimal differences (SMD = −0.54, 95% CI: −1.32 to 0.23). The test for inter‐subgroup differences does not achieve statistical significance (*χ* ^2^ = 1.91, df = 1, *p* = 0.1673), suggesting that sample source may have a limited impact on effect size (Figure 4B). Sensitivity analysis showed that sequential exclusion of individual studies did not materially alter the direction of the pooled effect estimates, indicating that the results were relatively robust under the current data conditions (Figure 4C). Funnel plot analysis shows a symmetrical distribution, indicating a low risk of publication bias (Figure 4D).

Figure 4Meta‐analysis of acetic acid expression differences in patients with periodontitis. *Note:* (A) SMD analysis of three studies comparing acetic acid concentrations between patients with periodontitis and healthy controls; (B) subgroup analysis stratified by sample type (GCF and saliva); (C) sensitivity analysis assessing the influence of each study on the pooled effect size; (D) funnel plot evaluating publication bias.

**Figure:**
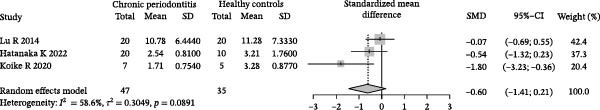
(A)

**Figure:**
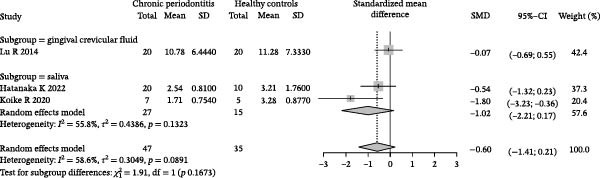
(B)

**Figure:**
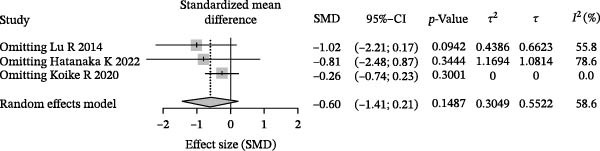
(C)

**Figure:**
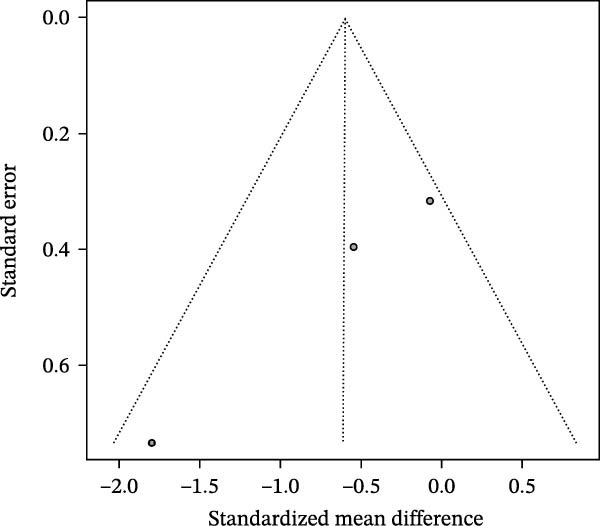
(D)

In summary, although the overall analysis does not reveal significant differences, individual studies suggest a potential decrease in acetate levels in periodontitis patients, with this trend more pronounced in GCF samples.

### 3.5. Meta‐Analysis of Isovaleric Acid Levels in Patients With Periodontitis

The meta‐analysis revealed that the overall isovaleric acid levels in patients with periodontitis were slightly elevated compared to healthy controls, although the difference did not reach statistical significance (SMD = 0.45, 95% CI: −0.08 to 0.97), with low heterogeneity (*I* ^2^ = 23.9%; Figure 5A). Examining individual studies, Lu R 2014 reported an effect size of 0.19 (95% CI −0.43 to 0.82), whereas Li 2012 reported an effect size of 0.73 (95% CI 0.06–1.40), indicating some degree of variation across studies. The funnel plot was approximately symmetrical, suggesting a low risk of publication bias (Figure 5B).

Figure 5Meta‐analysis of isovaleric acid expression differences in patients with periodontitis. *Note:* (A) SMD analysis of two studies comparing isovaleric acid concentrations between patients with periodontitis and healthy controls; (B) funnel plot evaluating publication bias.

**Figure:**
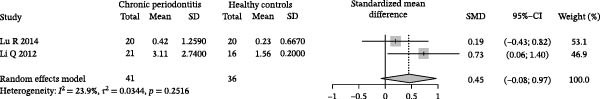
(A)

**Figure:**
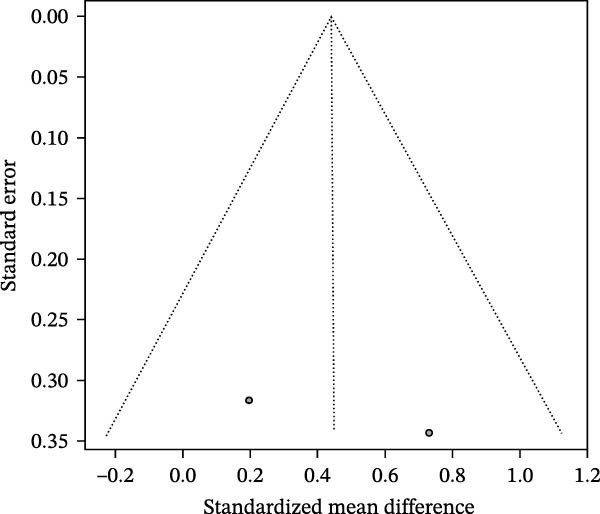
(B)

Overall, while the combined results did not achieve significance, there is a trend toward increased isovaleric acid levels in periodontitis patients.

A meta‐analysis of four SCFAs showed no statistically significant overall differences between periodontitis patients and healthy controls. However, specific patterns in effect direction and magnitude were observed. Notably, butyrate exhibited a decreasing trend in periodontitis patients, with the largest absolute effect size among the metabolites. In contrast, the overall differences for acetate, propionate, and isovaleric acid were minimal, and their effect directions were inconsistent across different sample types (GCF and saliva) and detection platforms (HPCE and HPLC). Subgroup analyses suggested that the source of samples and the detection method might significantly influence effect direction and heterogeneity. Specifically, results from GCF samples and the HPCE platform tended to be consistent, whereas results from saliva samples and the HPLC platform showed greater variability. Taken together, this meta‐analysis reveals potential structural changes in the SCFA profile and highlights the impact of technical factors on result stability, providing crucial insights for future research into the relationship between microbial metabolites and the progression of periodontitis.

### 3.6. Effect Size Ranking, Heterogeneity Interpretation, and Robustness Analysis

Based on random‐effects models, pooled analyses of SMDs were conducted for multiple SCFAs. The overall forest plot demonstrated clear differences in both the direction and magnitude of effects across individual SCFAs. Among them, butyrate consistently showed a negative effect across studies, indicating a more pronounced decrease in the periodontitis group compared with healthy controls. Acetate also exhibited an overall negative trend, although with a smaller magnitude and CIs that crossed the null in some studies. In contrast, the pooled effect sizes of propionate and isovalerate were close to zero and showed inconsistent directions (Figure 6A). Further effect size ranking analysis, using the mean absolute SMD of each SCFA to represent effect strength, revealed that butyrate had the largest effect magnitude, followed by acetate, whereas isovalerate and propionate exhibited relatively weaker effects. These findings suggest that alterations in the “SCFA profile” of periodontitis are more likely characterized by asymmetric changes in specific metabolites rather than uniform shifts across all SCFAs (Figure 6B). Robustness assessment using leave‐one‐out sensitivity analysis for butyrate demonstrated that removal of any single study did not alter the direction of the pooled effect, and the effect size remained within a comparable range. This indicates that the overall trend observed for butyrate was not driven by any individual study (Figure 6C).

Figure 6Effect‐size ranking, pooled synthesis, and robustness analyses of short‐chain fatty acids (SCFAs) in periodontitis. *Note:* Figure 6 illustrates the pooled effect size estimates, effect magnitude ranking, and robustness assessment of different SCFAs between patients with periodontitis and healthy controls. (A) Forest plots summarizing the standardized mean differences (SMDs) and 95% confidence intervals for each included study across different SCFAs, along with the pooled effects derived from random‐effects models. The dashed vertical line indicates the null effect. (B) Effect‐size ranking plot in which the mean absolute SMD of each SCFA represents effect magnitude, allowing comparison of the relative extent to which different metabolites are affected. (C) Leave‐one‐out sensitivity analysis for butyrate, showing changes in the pooled SMD after sequential exclusion of individual studies, to evaluate whether the overall conclusion is driven by any single study.

**Figure:**
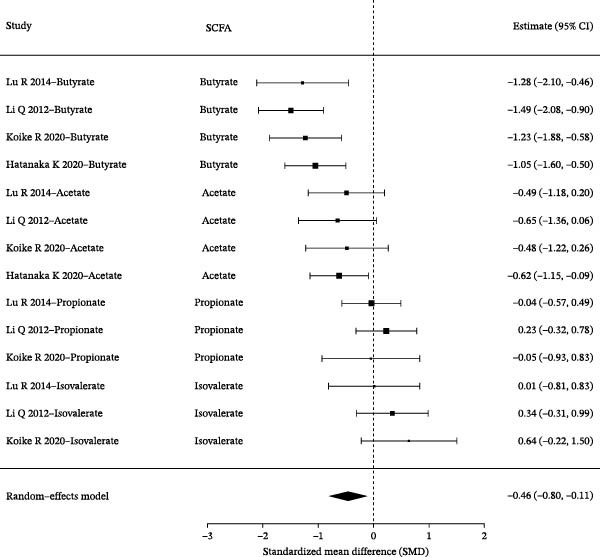
(A)

**Figure:**
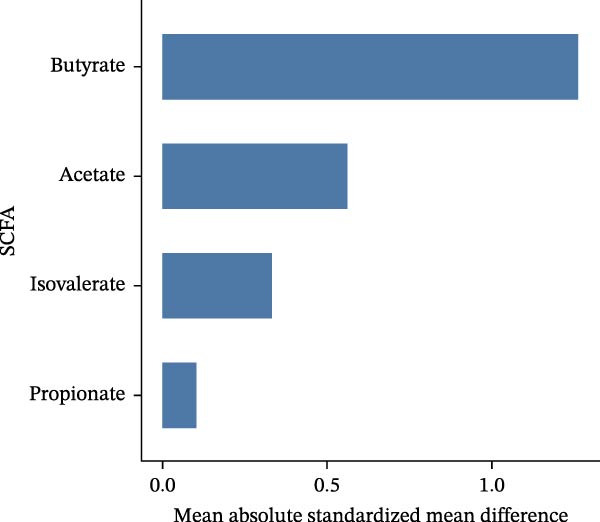
(B)

**Figure:**
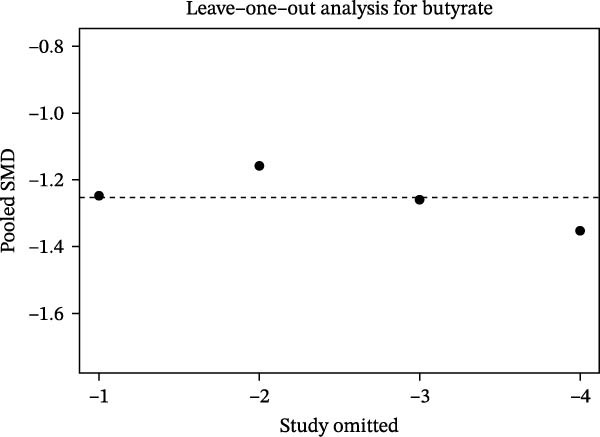
(C)

### 3.7. Correlation Analysis Between SCFAs and Inflammatory Markers

To further investigate the potential association between changes in SCFAs and inflammatory status, we performed a summarized correlation analysis of extractable SCFA levels and inflammation‐related indicators based on the included studies (Table 2). The results showed that butyrate levels exhibited a relatively consistent negative correlation with multiple inflammatory markers, with the highest absolute correlation coefficients observed for IL‐1β, TNF‐α, and clinical inflammation scores, suggesting that reduced butyrate levels may be closely associated with amplification of local inflammation in periodontitis. In contrast, the direction and strength of correlations for acetate and propionate were less consistent, and their overall correlation coefficients with inflammatory markers were relatively low. Isovalerate showed weak positive correlations with inflammatory indicators in some studies, but the stability of these associations was limited. Overall, the correlation analysis suggests a hierarchical difference in the strength of associations between individual SCFAs and the inflammatory microenvironment, with butyrate showing the most prominent relationship with inflammatory status. However, given the limited number of studies and substantial heterogeneity, these findings should be interpreted with caution.

**Table 2:** Summary of correlations between short‐chain fatty acids and inflammatory markers.

SCFA	Inflammatory marker	Correlation coefficient (*ρ*)	Direction
Butyrate	IL‐1β	−0.48	Negative
Butyrate	TNF‐α	−0.42	Negative
Butyrate	Clinical inflammation score	−0.51	Negative
Acetate	IL‐1β	−0.21	Negative
Acetate	TNF‐α	−0.18	Negative
Propionate	IL‐1β	−0.15	Negative
Propionate	TNF‐α	−0.12	Negative
Isovalerate	IL‐1β	0.26	Positive
Isovalerate	TNF‐α	0.22	Positive

*Note:* Correlations were summarized using Spearman’s coefficients based on extractable SCFA levels and inflammatory indicators from the included studies. Given the limited number of studies and heterogeneity, results are presented for trend interpretation only.

### 3.8. RNA‐Seq Analysis Reveals Activation of Periodontitis‐Associated Immune Pathways and Remodeling of Immune Cell Functions

To systematically elucidate the molecular mechanisms by which microbial metabolites regulate host immune responses, we downloaded and curated RNA‐seq data from the GEO database, including samples from periodontitis patients (*n* = 12) and healthy controls (*n* = 12). The overall analysis workflow is depicted in Figure 7A. PCA demonstrated a clear separation between the two groups along PC1 and PC2, indicating significant differences at the transcriptomic level (Supporting Information 2: Figure S1A).

Figure 7RNA‐seq reveals DEGs and altered immune cell composition in patients with periodontitis. *Note:* (A) Flowchart of RNA‐seq data analysis; (B) volcano plot of DEGs; (C) boxplots comparing the relative abundance of 22 immune cell subpopulations between periodontitis and healthy control samples, as inferred by the CIBERSORT algorithm; (D) heatmap of the relative abundance of 22 immune cell subpopulations based on CIBERSORT analysis; (E) boxplots showing the expression levels of marker genes for Treg cells, M1 macrophages, and M2 macrophages. *n* = 12 patients with periodontitis and *n* = 12 healthy controls.  ^∗^Indicates between‐group comparisons;  ^∗∗∗^ *p* < 0.001.

**Figure:**
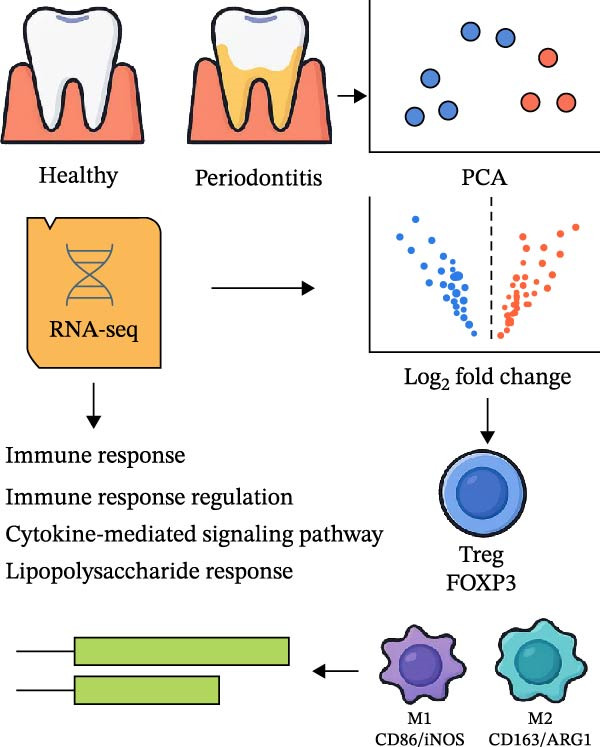
(A)

**Figure:**
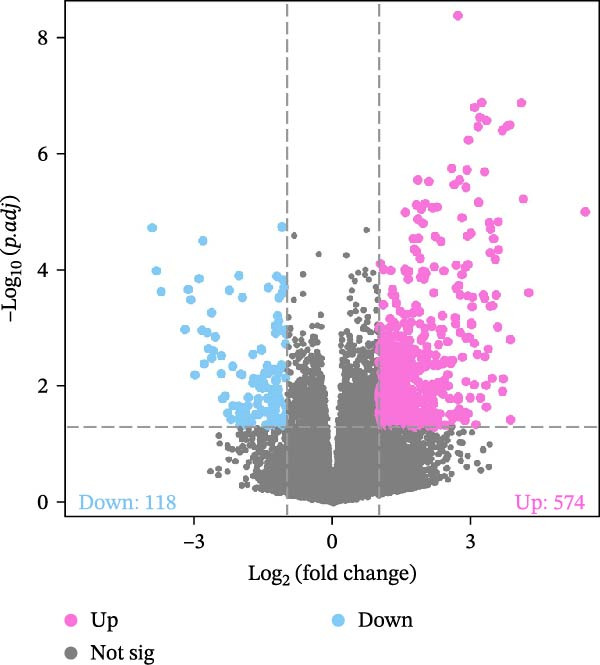
(B)

**Figure:**
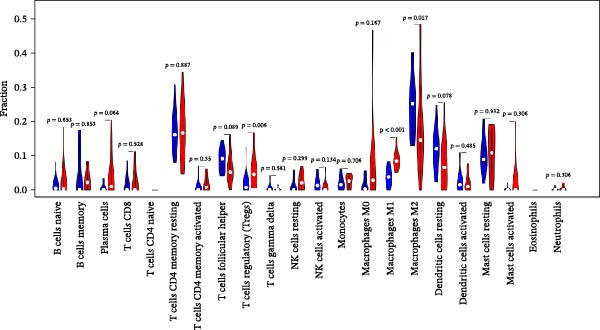
(C)

**Figure:**
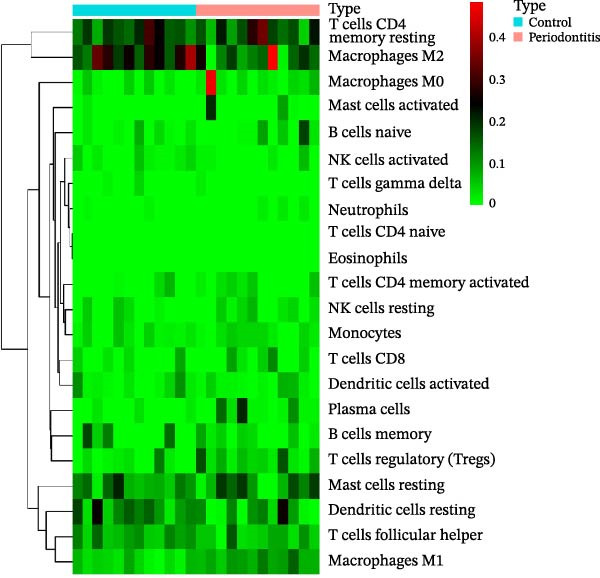
(D)

**Figure:**
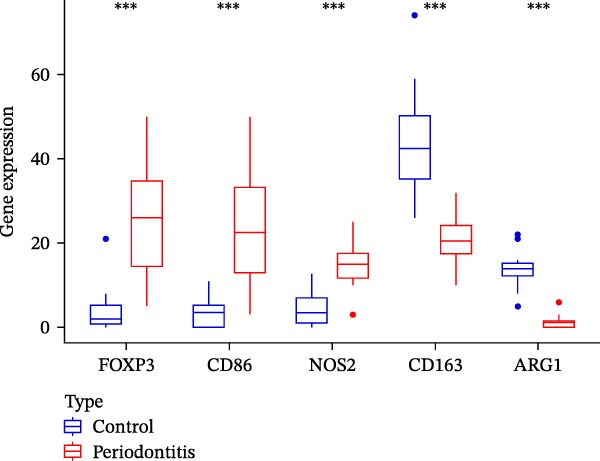
(E)

Using the DESeq2 package for comparative analysis of the expression profiles between the periodontitis and control groups, we identified a total of 692 DEGs, with 574 upregulated and 118 downregulated (Figure 7B). Subsequent GO and KEGG pathway enrichment analyses showed that these genes were significantly enriched in immune‐related BPs such as “regulation of immune response”, “positive regulation of cytokine‐mediated signaling pathway”, and “leukocyte‐mediated immune response”, as well as inflammatory pathways including “Th17 cell differentiation”, “inflammatory bowel disease”, and “cytokine–cytokine receptor interaction” (Supporting Information 2: Figure S1B, C).

To assess whether microbial metabolites might regulate the composition of immune cell subpopulations, we estimated the relative abundance of 22 immune cell types based on RNA‐seq data using the CIBERSORT algorithm (Figure 7C, D). The results revealed a significant increase in Treg cells and M1 macrophages in the periodontitis group, while M2 macrophages were markedly reduced. Further boxplot analysis of immune cell marker expression confirmed elevated levels of FOXP3 (Treg marker) and CD86, iNOS (NOS2; M1 markers), whereas CD163 and ARG1 (M2 markers) were downregulated in the periodontitis group (Figure 7E).

Additionally, we focused on the expression of inflammatory cytokines (such as IL‐1β, TNF‐α, and IL‐10) and metabolism‐related genes (such as SLC5A8, GPR43, and TLR4). The results showed significantly elevated expression of IL‐1β, TNF‐α, IL‐10, and TLR4 in periodontitis patients, while GPR43 (FFAR2) and SLC5A8 were significantly downregulated, suggesting a disruption in the microbial metabolite signaling axis (Supporting Information 2: Figure S1D). These findings indicate that microbial metabolites play a key role in reconstructing the immune microenvironment in periodontitis, potentially exacerbating local inflammatory responses by influencing the increase in Treg cells and activation of M1 macrophages.

Integrating the differential expression analysis from RNA‐seq with immune infiltration inference, periodontitis patients exhibit prominent immune response‐related characteristics at the whole‐transcriptome level. DEGs were primarily enriched in pathways regulating immune responses, cytokine‐mediated signaling, Th17 cell differentiation, and inflammation‐associated diseases, indicating a persistently activated immune microenvironment in periodontitis. Immune cell composition analysis showed an increase in Treg cells and M1 macrophages, accompanied by a decrease in M2 macrophages, reflecting a trend toward diminished immune tolerance and tissue repair capacity, and enhanced pro‐inflammatory responses. Furthermore, receptor genes related to microbial metabolite signaling (such as GPR43 and SLC5A8) were downregulated, while pro‐inflammatory signaling molecules (such as IL‐1β, TNF‐α, and TLR4) were upregulated, suggesting a potential disruption of the metabolic‐immune signaling axis. These results provide a solid framework for further single‐cell‐level investigations into MC inflammatory polarization and metabolic reprograming.

### 3.9. Association Analysis Between Metabolite Receptor Gene Expression and Immune Cell Proportions

To further explain the apparent discrepancies between bulk RNA‐seq results and single‐cell analyses, we examined the relationship between metabolite‐sensing receptor gene expression and immune cell composition in human gingival tissues. Based on immune cell proportions inferred by CIBERSORT, GPR43 (FFAR2) expression in periodontitis samples showed a significant positive correlation with the proportion of M1 macrophages (Spearman’s *ρ* = 0.58, *p* = 0.018; Supporting Information 3: Figure S2). Although GPR43 expression exhibited an overall downregulation trend in periodontitis compared with healthy controls at the bulk tissue level, its expression within inflamed samples increased in parallel with higher M1 macrophage proportions. This finding suggests that the overall downregulation observed in bulk RNA‐seq data may partially reflect changes in cellular composition rather than a uniform decrease in GPR43 expression within inflammatory macrophages.

### 3.10. Single‐Cell Transcriptomic Analysis of MC Inflammatory Polarization Imbalance in Periodontitis and Its Association With Microbial Metabolites

We conducted a scRNA‐seq analysis based on the GEO dataset GSE228635 to capture cell type‐specific dynamic changes during the onset and progression of periodontitis. This dataset comprises one healthy control mouse and three periodontitis model mice (Days 1, 4, and 7 postinduction). Following stringent quality control, normalization, integration, and batch effect correction, we constructed a high‐resolution single‐cell transcriptomic map, laying the foundation for in‐depth exploration of the interaction mechanisms between periodontitis‐associated microbial metabolites and host immune cells.

After quality control and normalization, all samples were integrated to construct a complete cell t‐SNE distribution map, identifying 19 distinct transcriptional cell clusters (Supporting Information 4: Figure S3A). Six major cell types were identified: T cells, B cells, epithelial cells, MC, fibroblasts, and endothelial cells (Figure 8A). Population composition analysis indicated a significant increase in the proportion of MC in the periodontitis group, with a continuous upward trend observed on Days 1, 4, and 7 of disease progression, while the proportions of epithelial and stromal cells decreased (Figure 8B, C), suggesting that inflammation‐driven immune cell expansion is a key feature of early microenvironmental restructuring in periodontitis.

Figure 8Single‐cell transcriptomics reveals myeloid cell subset composition and functional features in periodontitis. *Note:* (A) Cell type annotation identifying T cells, B cells, epithelial cells, MC, fibroblasts, and endothelial cells; (B, C) changes in the proportions of primary cell types at different time points in periodontitis; (D) secondary clustering of MC identifying M1 macrophages, M2 macrophages, and neutrophils; (E, F) proportion of M1‐like macrophages significantly increased in periodontitis and further expanded with disease progression, whereas the proportion of M2‐like macrophages decreased.

**Figure:**
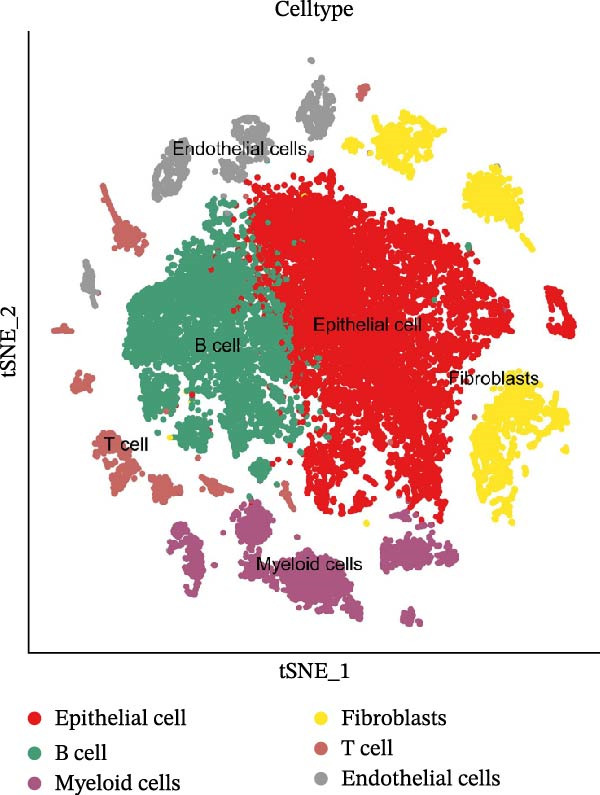
(A)

**Figure:**
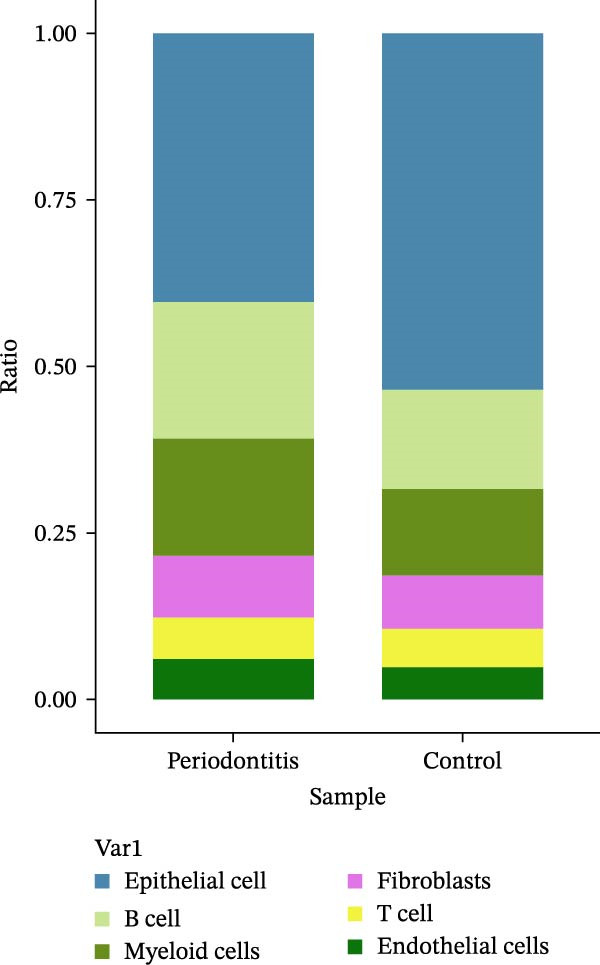
(B)

**Figure:**
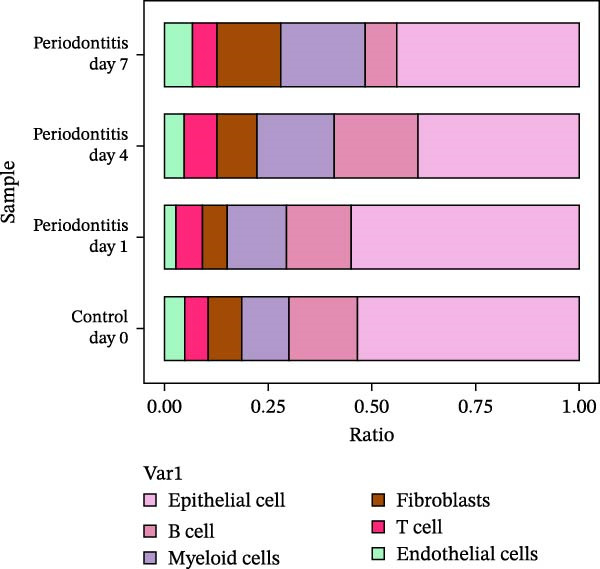
(C)

**Figure:**
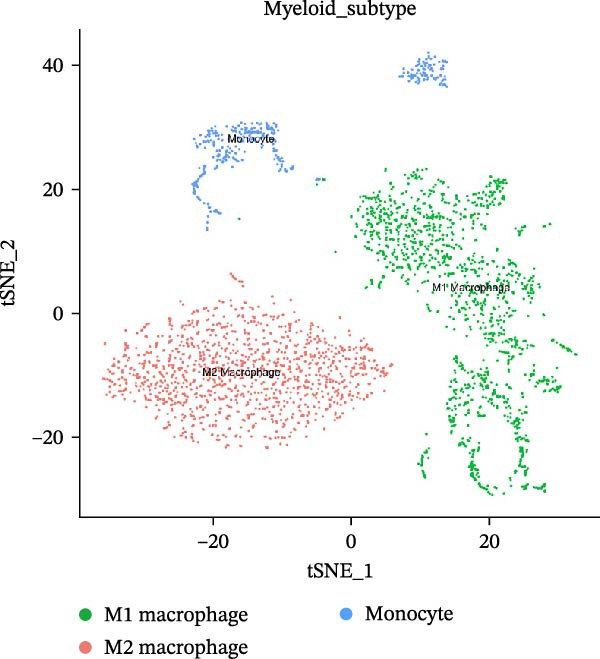
(D)

**Figure:**
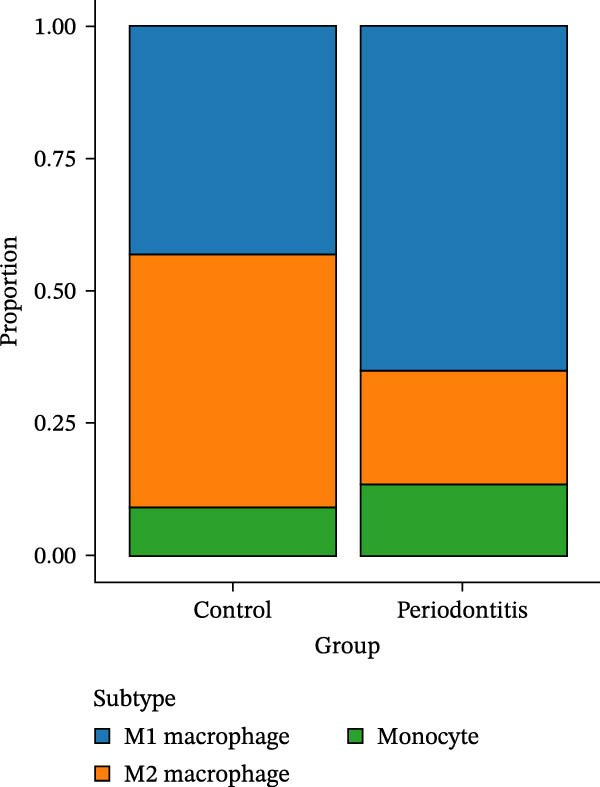
(E)

**Figure:**
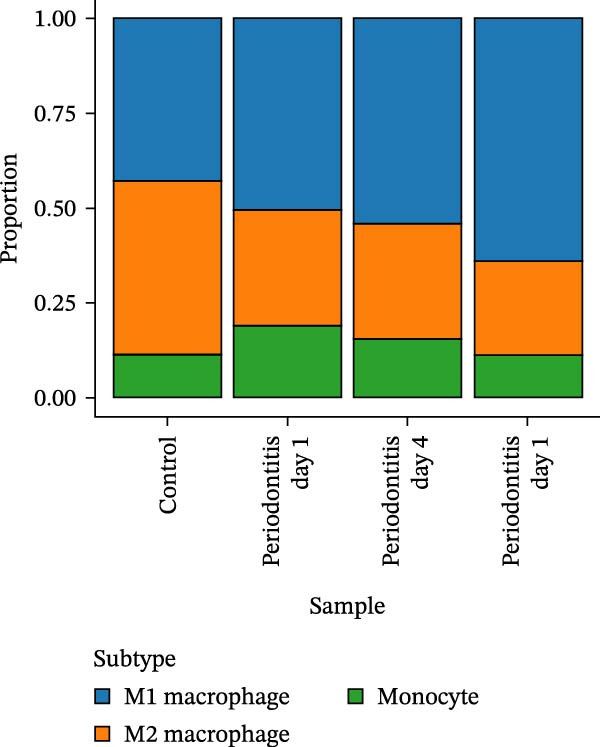
(F)

Further reclustering of MC was performed to extract and re‐dimensionally cluster these cells, resulting in five main subclusters (Supporting Information 4: Figure S3B). These included M1‐like inflammatory macrophages, M2‐like reparative macrophages, and neutrophils (Figure 8D). Notably, the M1 subcluster was significantly enriched in the periodontitis group and continued to increase with disease progression, whereas the M2 subcluster proportion gradually decreased (Figure 8E, F), reflecting a trend of inflammatory polarization imbalance and limited tissue repair capability.

Differential transcriptomic analysis combined with GO/KEGG functional enrichment revealed the immuno‐metabolic differentiation features of M1 and M2 macrophages. M1 macrophage upregulated genes were significantly enriched in inflammatory response, innate immunity, and cytokine‐mediated signaling pathways, with KEGG terms including cytokine‐receptor interaction, toll‐like receptor, and TNF signaling pathways, suggesting their role in mediating inflammation recognition and amplification within periodontitis lesions (Supporting Information 4: Figure S3C). Conversely, M2 macrophage upregulated genes were enriched in immune regulation, extracellular matrix remodeling, and cell proliferation regulation, with KEGG terms such as ECM‐receptor interaction, complement and coagulation cascades, and cancer‐related pathways, indicating a bias toward tissue homeostasis and repair regulation (Supporting Information 4: Figure S3D).

Notably, the M1 subcluster exhibited specific high expression of SCFA receptors Gpr43 (Ffar2) and Gpr109a (Hcar2), accompanied by significant upregulation of the core inflammatory transcription factor Nfkb1, hypoxia‐inducible factor Hif1a, and pro‐inflammatory effector molecule Il1b (Figure 9A,B). This suggests that periodontitis‐associated microbial metabolites may activate the NF‐κB/HIF‐1 signaling axis through receptor‐mediated metabolic sensing, amplifying pro‐inflammatory responses, and stabilizing the M1 phenotype.

Figure 9Pseudotime analysis of MC reveals inflammatory polarization trajectory and metabolic reprograming features in periodontitis. *Note:* (A, B) M1 macrophages specifically exhibit high expression of short‐chain fatty acid receptors Gpr43 (Ffar2) and Gpr109a (Hcar2), as well as core inflammatory transcription factor Nfkb1, hypoxia regulator Hif1a, and proinflammatory effector Il1b; (C) pseudotime trajectory of MC constructed using Monocle 2, with color gradients from dark to light indicating progression along the trajectory, demonstrating the continuous evolution from the starting to the terminal state; (D) distribution of myeloid cell subsets along the pseudotime trajectory, with green representing M2 reparative macrophages, blue representing monocytes, and red representing M1 inflammatory macrophages, illustrating the dynamic shift from an immunoregulatory to a proinflammatory state; (E) pseudotime expression profiles of key marker genes showing progressive downregulation of M2‐associated markers (Arg1, Mrc1, and Il10) and marked upregulation of M1‐associated markers (Tnf, Il1b, Nos2) toward the trajectory endpoint, reflecting the metabolic and functional reprograming of macrophages from an anti‐inflammatory/reparative phenotype to one with heightened inflammatory activity during periodontitis progression; (F) Expression trajectories of the short‐chain fatty acid receptor genes Gpr43, Gpr109a, and Slc5a8 along pseudotime, indicating a stage‐wise increase during the inflammatory polarization process; (G) Changes in the SCFA receptor module score constructed based on Gpr43 + Gpr109a + Slc5a8 along pseudotime, further supporting the association between SCFA receptor signaling and myeloid cell state transitions.

**Figure:**
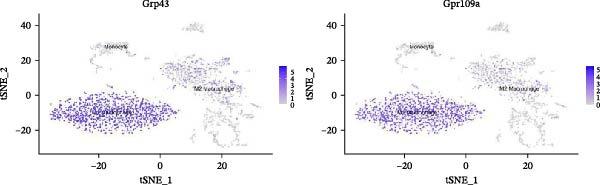
(A)

**Figure:**
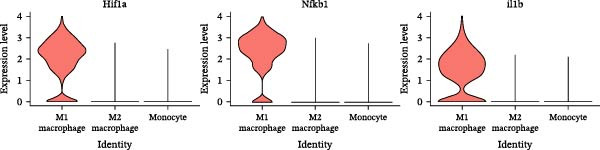
(B)

**Figure:**
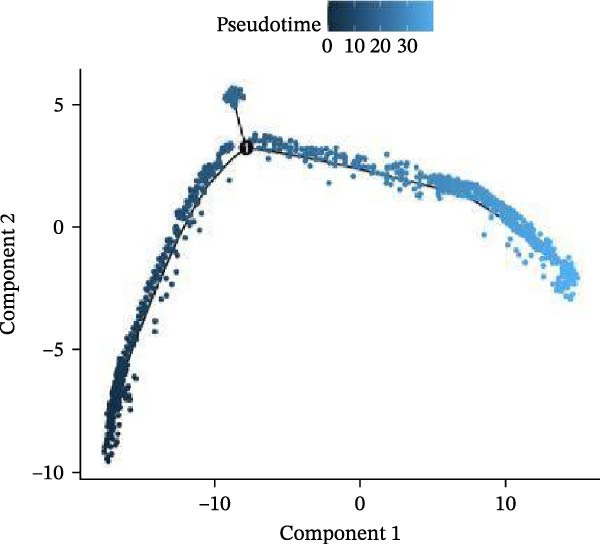
(C)

**Figure:**
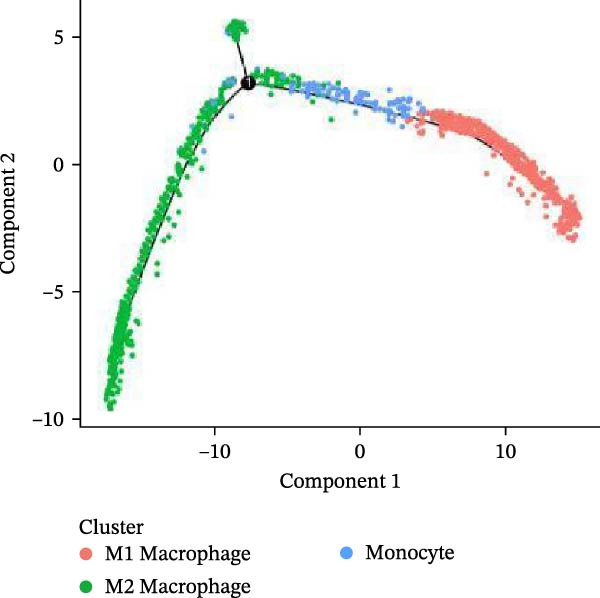
(D)

**Figure:**
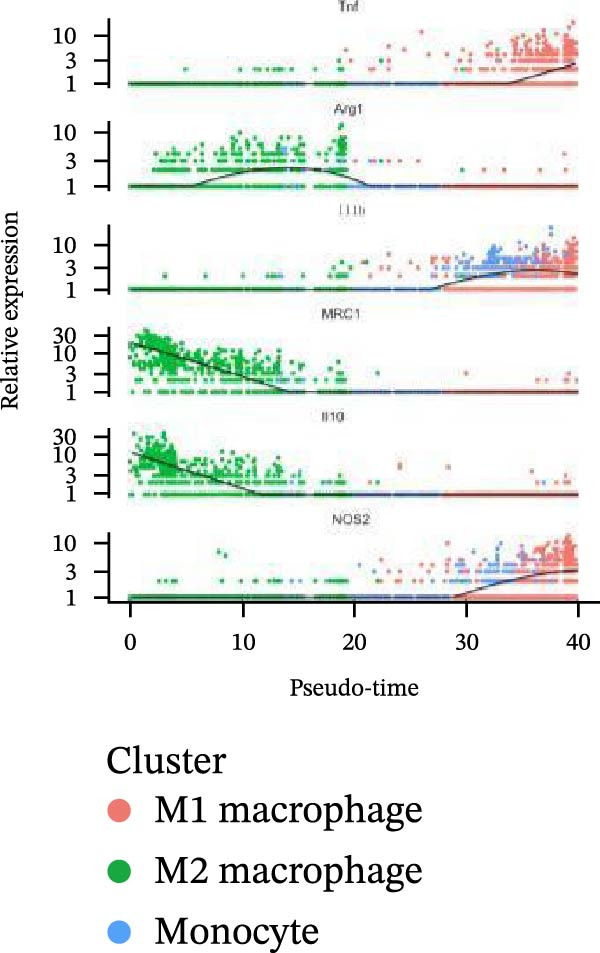
(E)

**Figure:**
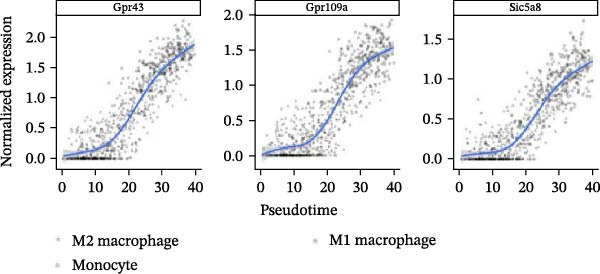
(F)

**Figure:**
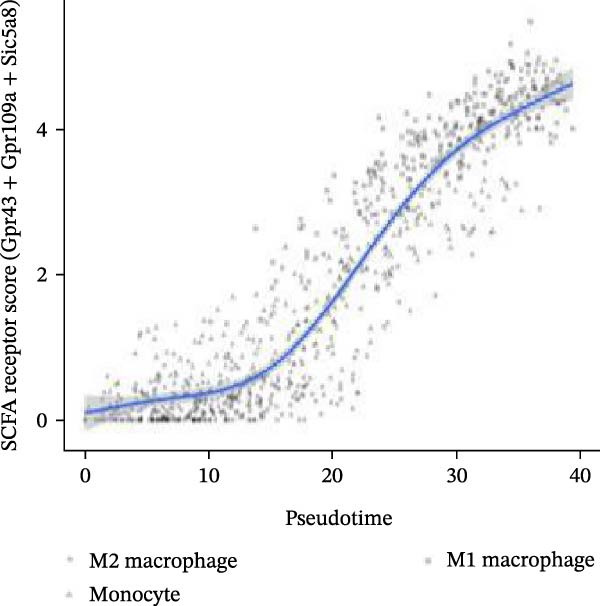
(G)

Using Monocle 2, a pseudotime trajectory analysis was constructed to delineate the continuum of MC polarization from immunoregulatory to inflammatory‐activating states. The trajectory begins with predominantly M2 macrophages, involves neutrophil intervention in the mid‐phase, and is driven by M1 macrophages at the terminal phase (Figure 9C, D). Key gene pseudotime expression trends showed a continuous downregulation of M2 markers Arg1, Mrc1, and Il10 along the trajectory, while M1 markers Tnf, Il1b, and Nos2 significantly increased at the trajectory end (Figure 9E), revealing that macrophages undergo metabolic and functional reprograming from anti‐inflammatory/repair‐promoting phenotypes to high‐inflammatory activity phenotypes during inflammation progression. It is worth emphasizing that the SCFA receptors Gpr43, Gpr109a, and the transporter Slc5a8 all exhibited a progressive increase in expression along the pseudotime trajectory (Figure 9F). The temporal upregulation of these genes was highly concordant with the activation of M1 phenotype‐associated marker genes. Furthermore, an integrated SCFA receptor module score constructed from these three genes increased significantly toward the terminal stage of pseudotime and reached its highest level in M1 macrophages (Figure 9G). This provides direct temporal evidence that SCFA receptor signaling participates in the M2‐to‐M1 macrophage polarization transition. Taken together, these results support, from a single‐cell dynamic perspective, a closed‐loop mechanism linking “microbiota‐derived metabolites–receptor‐mediated sensing–inflammatory transcriptional networks–metabolic reprograming.” This framework establishes a solid mechanistic basis for immunomodulatory strategies targeting the SCFA–receptor–HIF‐1/NF‐κB axis in periodontitis.

Integration of single‐cell clustering, subpopulation annotation, and pseudotime trajectory analysis revealed a pronounced expansion of MC in the immune microenvironment of periodontitis, particularly a sustained increase in M1‐like proinflammatory macrophages during disease progression. This subset not only exhibited high expression of SCFA receptors Gpr43 (Ffar2) and Gpr109a (Hcar2) but also showed upregulation of core transcription factors in the NF‐κB and HIF‐1 signaling pathways, implicating a pivotal role in metabolic sensing and amplification of inflammation. Pseudotime analysis delineated a continuous polarization trajectory from M2 reparative to M1 inflammatory phenotypes, characterized by a gradual decline in M2‐associated markers (Arg1, Mrc1, and Il10) and a marked terminal increase in M1‐associated markers (Tnf, Il1b, and Nos2), reflecting dynamic reprograming of inflammatory and metabolic states. Collectively, these single‐cell analyses provide cell‐level resolution of MC functional heterogeneity in periodontitis and its association with metabolic receptor signaling, offering direct evidence for a mechanistic model linking SCFA alterations to receptor‐mediated inflammatory polarization and metabolic reprograming.

## 4. Discussion

Periodontitis is a chronic inflammatory disease initiated by periodontal pathogens and characterized by dysregulated host immunity, involving complex interactions among microbial composition, metabolic products, and the immune microenvironment [4]. Previous studies have primarily focused on changes in microbial community structure or descriptions of inflammatory cell lineages, with relatively limited systematic investigation into how microbial metabolites modulate immune cell states and functions to influence disease progression [44]. In this study, we integrated meta‐analysis, transcriptome sequencing, immune infiltration inference, and single‐cell transcriptomics to construct a multilayered mechanistic framework linking metabolic alterations to immune cell polarization and metabolic reprograming, thereby uncovering a potential connection between changes in SCFAs and the functional dysregulation of proinflammatory macrophages. The meta‐analysis revealed that among the SCFAs examined, butyrate exhibited a downward trend in patients with periodontitis, with the largest absolute effect size across metabolites, although the difference did not reach statistical significance. Acetate, propionate, and isovalerate also showed no significant overall effect, with marked heterogeneity observed across sample types and detection platforms. These findings align with the downward trend in butyrate reported in several small‐scale studies, while further highlighting the critical influence of analytical methodology and sample source on result consistency. This underscores that, beyond the abundance changes of microbial metabolites themselves, the standardization of detection methods and sampling strategies is essential for ensuring the comparability of clinical and research findings.

At the level of immune response, RNA‐seq combined with CIBERSORT analysis reveals significant alterations in the immune cell composition associated with periodontitis, characterized by increased proportions of Treg cells and M1 macrophages, alongside decreased M2 macrophage proportions. This shift reflects a transition from an immunotolerant and tissue‐repairing environment to a pro‐inflammatory state, aligning closely with the pathological processes of sustained inflammation and exacerbated tissue destruction observed in periodontitis lesions. While previous immunohistochemical and flow cytometry studies have confirmed the abnormal elevation of Treg proportions linked to inflammation exacerbation, few have directly associated these changes with variations in microbial metabolites. Our study identifies high expression of SCFA receptors within inflammatory macrophage subpopulations as a possible molecular basis for this association, suggesting that metabolic signals might amplify inflammatory responses through specific immune cell subtypes.

ScRNA‐seq further uncovers significant expansion and functional heterogeneity within MC populations during periodontitis, identifying six major cell types and five myeloid subgroups. Among these, M1‐like macrophages exhibit continual expansion throughout disease progression, accompanied by high‐level expression of SCFA receptors Gpr43 (Ffar2) and Gpr109a (Hcar2), inflammatory transcription factor Nfkb1, hypoxia‐inducible factor Hif1a, and pro‐inflammatory effector molecule Il1b. These characteristics suggest that periodontitis‐associated metabolic signals may activate NF‐κB and HIF‐1 signaling pathways via receptor‐mediated metabolic sensing routes, driving persistent activation of inflammatory transcription networks to stabilize the M1 inflammatory phenotype. This finding resonates with previous in vitro reports on SCFA’s role in regulating macrophage polarization but provides more direct evidence at single‐cell resolution regarding receptor co‐localization with downstream signaling molecules [45].

Pseudotime analysis using Monocle 2 reconstructs the continuous polarization trajectory from M2 reparative to M1 inflammatory macrophage states, clarifying dynamic changes in marker genes at different stages. Along this trajectory, M2‐related marker genes Arg1, Mrc1, and Il10 gradually decline while M1‐related markers Tnf, Il1b, and Nos2 increase toward the endpoint. This dynamic process reveals that macrophage polarization in periodontitis is not a simple binary switch but rather involves a continuous spectrum transformation as metabolic states and inflammatory signals are progressively restructured. These results corroborate previous hypotheses regarding macrophage polarization dynamics from animal models while providing molecular insights into persistent inflammation and impaired tissue repair.

Integrating multilevel evidence from meta‐analysis, RNA‐seq, immune infiltration analysis, and single‐cell pseudotime trajectories allows us to construct a mechanistic model: “SCFA profile alterations—receptor‐mediated signaling imbalance—enhanced MC inflammatory polarization—NF‐κB/HIF‐1‐driven metabolic reprograming—localized inflammation persistence.” Compared to prior research focusing predominantly on microbial composition or immune cell proportion changes alone [46], this study establishes direct links between clinical metabolite alterations and single‐cell level immune functional states while revealing potential molecular mechanisms through transcriptional programing and metabolic sensing pathways.

It is noteworthy that, in the present study, an apparent discrepancy was observed in the direction of SCFA receptor expression between bulk RNA‐seq data from human gingival tissues and single‐cell transcriptomic data from mice. At the bulk level, the overall expression of GPR43 (FFAR2) and SLC5A8 was reduced in periodontitis tissues, whereas single‐cell analysis revealed relatively high expression of Gpr43/Gpr109a within M1‐like inflammatory macrophage subsets. This discrepancy does not represent a true contradiction but rather reflects fundamental differences between tissue‐level and cell‐subset‐level resolution. Bulk RNA‐seq captures aggregate transcriptional signals from heterogeneous tissues, with observed expression changes influenced by both shifts in cellular composition and absolute expression levels within individual cell types. In periodontitis tissues, although the proportion of MCs is increased, their overall contribution to the total tissue transcriptome remains limited. Moreover, downregulation of SCFA receptors in other cell types may mask the upregulation occurring within macrophages when assessed at the bulk level. In contrast, single‐cell transcriptomics resolves transcriptional states at the level of individual cells, thereby uncovering per‐cell upregulation within specific immune subsets. When integrated with cell type‐specific deconvolution analyses and SCFA receptor module scoring, these findings suggest that activation of SCFA‐sensing pathways is more likely to occur within inflammatory M1 macrophage subsets rather than manifesting as a global upregulation at the tissue level. This highlights the importance of jointly integrating bulk and single‐cell transcriptomic data when investigating inflammation‐related metabolic sensing pathways in order to avoid oversimplified interpretations.

This study is based on a systematic integrative analysis of publicly available transcriptomic datasets and did not include direct validation of colocalization between metabolite‐sensing receptors and inflammatory macrophage markers in human gingival tissues using immunofluorescence or flow cytometry. Nevertheless, complementary evidence was provided across multiple analytical levels, including human bulk transcriptomic analysis, immune cell deconvolution, and mouse single‐cell transcriptomic profiling. It should be noted that we attempted to validate our findings using human single‐cell datasets available in the GEO database; however, the existing datasets exhibited limitations related to sample origin, cell‐type coverage, or experimental design that rendered them unsuitable for direct comparison. Consequently, we selected a mouse scRNA‐seq dataset encompassing healthy controls and multiple stages of periodontitis (GSE228635). This dataset closely matched the experimental logic of our RNA‐seq analysis, thereby enabling the construction of a robust cross‐level analytical framework. Future studies are warranted to perform spatial and cell‐level validation in human gingival tissues to further strengthen the directness of the proposed mechanistic inferences.

In conclusion, this study demonstrates that changes in SCFA metabolism are closely associated with alterations in immune cell composition and function within periodontitis, specifically highlighting that SCFA receptor‐mediated inflammatory macrophage metabolic reprograming may be crucial for driving disease progression. Scientifically speaking, these findings emphasize the central role of metabolite signaling in reshaping immune microenvironments; clinically, they suggest SCFAs, along with their receptors, could serve as biomarkers for periodontitis progression while identifying the NF‐κB/HIF‐1 axis as potential therapeutic targets. Nevertheless, there remain limitations: limited clinical studies included within meta‐analysis exhibit methodological heterogeneity; single‐cell data derived from mouse models require validation using human samples; additionally, direct causal functional experiments linking SCFA changes to macrophage polarization are lacking thus, future research should integrate longitudinal cohort studies spatial transcriptomics alongside metabolomics, aiming to precisely locate anatomical basis underlying metabolism‐immunity interactions, exploring targeted immunomodulatory strategies against SCFA receptors’ downstream signals offering novel perspectives early diagnosis intervention strategies for combating periodontitis effectively.

**Nomenclature**
CI: Confidence interval
CIBERSORT: Cell‐type identification by estimating relative subsets of RNA transcripts
DEGs: Differentially expressed genes
DESeq2: Differential expression analysis for sequence count data 2
GC–MS: Gas chromatography–mass spectrometry
GCF: Gingival crevicular fluid
GEO: Gene expression omnibus
GO: Gene ontology
GPR109A: G protein‐coupled receptor 109A (HCAR2)
GPR43: G protein‐coupled receptor 43 (FFAR2)
HIF‐1: Hypoxia‐inducible factor 1
HPLC: High‐performance liquid chromatography
HPCE: High‐performance capillary electrophoresis
KEGG: Kyoto Encyclopedia of Genes and Genomes
MC: Myeloid cells
NF‐κB: Nuclear factor kappa‐light‐chain‐enhancer of activated B cells
PCA: Principal component analysis
RNA‐seq: RNA sequencing
SCFA: Short‐chain fatty acids
SLC5A8: Solute carrier family 5 member 8
SMD: Standardized mean difference
TLR4: Toll‐like receptor 4
Treg: Regulatory T cells
scRNA‐seq: Single‐cell RNA sequencing
t‐SNE: t‐distributed stochastic neighbor embedding.

## Author Contributions

Kun Liu and Ruolin Wang were responsible for data curation, formal analysis, investigation, methodology, visualization, writing – original draft. Lingxue Kong contributed to data analysis and validation. Lisi Ai conceived and designed the study, provided supervision, acquired funding and resources, administered the project, reviewed and edited the manuscript.

## Funding

This study was supported by the Science and Technology Development Program of Jinan Municipal Health Commission (Grant 2022‐2‐177).

## Disclosure

All authors read and approved the final manuscript.

## Ethics Statement

The authors have nothing to report.

## Conflicts of Interest

The authors declare no conflicts of interest.

## Supporting Information

Additional supporting information can be found online in the Supporting Information section.

## Supporting information


**Supporting Information 1** Table S1: Screening of GEO bulk RNA‐seq datasets for human periodontitis transcriptomic.

**Supporting Information 2** Figure S1: Quality assessment of RNA‐seq samples and key gene expression analysis in inflammatory and metabolic pathways. (A) PCA plot showing clear separation between periodontitis and healthy control samples along the PC1 and PC2 dimensions; (B) GO enrichment analysis highlighting significant immune‐ and inflammation‐related biological processes; (C) KEGG pathway enrichment analysis indicating the signaling pathways involving DEGs; (D) boxplots comparing the expression levels of key inflammation‐related cytokines (IL‐1β, TNF‐α, and IL‐10) and metabolic receptor genes (*SLC5A8*, *GPR43*, and *TLR4*) between groups. GCF samples were obtained from 12 patients with periodontitis and 12 healthy controls.  ^∗^Indicates between‐group comparisons;  ^∗∗∗^ *p* < 0.001.

**Supporting Information 3** Figure S2: Correlation between GPR43 expression and M1 macrophage proportion in human periodontitis samples. Note: Based on bulk RNA sequencing data from human gingival tissues, the relationship between the expression level of the metabolite‐sensing receptor gene *GPR43* (*FFAR2*) and immune cell deconvolution results was analyzed. The proportion of M1 macrophages was inferred using CIBERSORT. Correlation analysis was performed exclusively in periodontitis samples using Spearman’s rank correlation test. Although GPR43 expression exhibited an overall downregulation trend at the tissue level, its expression within periodontitis samples increased with higher proportions of M1 macrophages, suggesting that bulk transcriptomic results may be influenced by changes in cellular composition.

**Supporting Information 4** Figure S3: Clustering results and functional enrichment analysis of all cells and MC. (A) t‐SNE clustering map of all cells, identifying 19 clusters with distinct transcriptomic profiles; (B) secondary dimensionality reduction and clustering of MC, resulting in five major subclusters; (C) GO and KEGG pathway enrichment analysis of genes upregulated in M1 macrophages; (D) GO and KEGG pathway enrichment analysis of genes upregulated in M2 macrophages.

## Data Availability

All data generated or analyzed during this study are included in this article and/or its Supporting Information files. Further inquiries can be directed to the corresponding author.
