# Supplementary material for: Signaling Through SCFA Receptors Gpr43 and Gpr109a Drives Pro‐Inflammatory M1 Macrophage Polarization in Periodontitis
Source: Mediators Inflamm. 2026 Apr 8;2026:3542645. doi: 10.1155/mi/3542645 (PMC13058726; doi:10.1155/mi/3542645)
Supplement: Supplementary file 1 — Supporting Information 1 Table S1: Screening of GEO bulk RNA‐seq datasets for human periodontitis transcriptomic. [file MI-2026-3542645-s003.docx]

**Table S1. Screening of GEO bulk RNA-seq datasets for human periodontitis transcriptomic analysis**

| **GEO accession** | **Sample type** | **Study design** | **Platform** | **Decision** | **Reason** |
| --- | --- | --- | --- | --- | --- |
| **GSE173082** | Human gingival tissue | Baseline transcriptomic profiling of periodontitis vs healthy controls | RNA-seq (Illumina) | **Included** | Primary human gingival tissues with clear disease/control grouping, untreated baseline samples, and sufficient transcriptomic depth for differential expression, immune deconvolution, and pathway analysis |
| GSE307844 | THP-1 and U937 monocytic cell lines | In vitro exposure to enamel matrix derivative (EMD) ± TGF-β receptor inhibitor | Illumina HiSeq 2000 | Excluded | In vitro monocytic cell line model; lacks primary gingival tissue and clinical periodontitis vs healthy comparison |
| GSE297378 | Human oral keratinocytes (HOKs) | In vitro stimulation with acetylcholine and Porphyromonas gingivalis | Illumina NovaSeq 6000 | Excluded | Epithelial cell culture under acute stimulation; does not represent baseline gingival tissue transcriptome from patients |
| GSE269628 | Gingival mesenchymal stem cells (G-MSCs) | In vitro stimulation with oxLDL or inflammatory cytokine cocktail | Illumina MiSeq | Excluded | Isolated stem cell population under experimental inflammatory conditions; not whole gingival tissue |
| GSE288367 | Gingival epithelial cell line (TIGK) | In vitro exposure to Streptococcus sanguinis extracellular membrane vesicles | Illumina NovaSeq 6000 | Excluded | Cell line–based host–microbe interaction model; lacks clinical disease vs control tissue samples |
| GSE273165 | Human gingival tissue | RNA-seq of severe periodontitis vs healthy controls | Illumina NovaSeq 6000 | Excluded | Limited sample size and focused on autophagy-related mechanisms; insufficient for robust immune deconvolution and multi-pathway analysis |

Note: The GEO database was systematically screened for human RNA-seq datasets related to periodontitis. Inclusion criteria were human gingival tissue samples, RNA sequencing, and clearly defined periodontitis and healthy control groups. In vitro cell line–based studies, experimentally stimulated samples, and datasets unsuitable for immune deconvolution were excluded. Only GSE173082 met all inclusion criteria and was therefore selected for bulk RNA-seq analysis.
