# Supplementary figures and images for: Signaling Through SCFA Receptors Gpr43 and Gpr109a Drives Pro‐Inflammatory M1 Macrophage Polarization in Periodontitis
Source: Mediators Inflamm. 2026 Apr 8;2026:3542645. doi: 10.1155/mi/3542645 (PMC13058726; doi:10.1155/mi/3542645)

**A**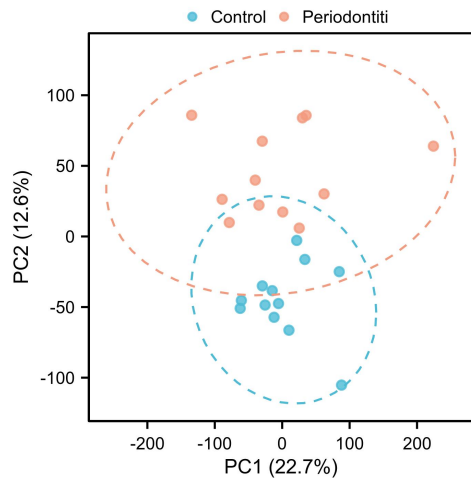**B**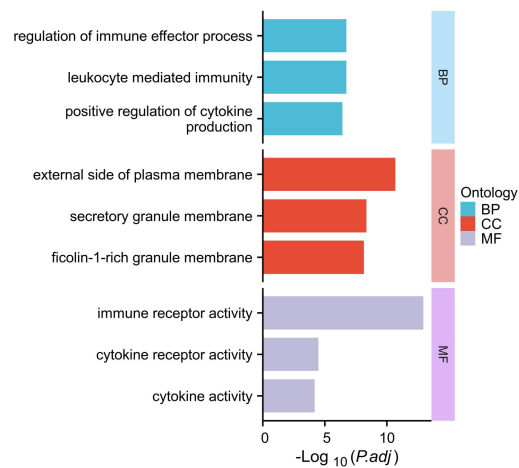**C**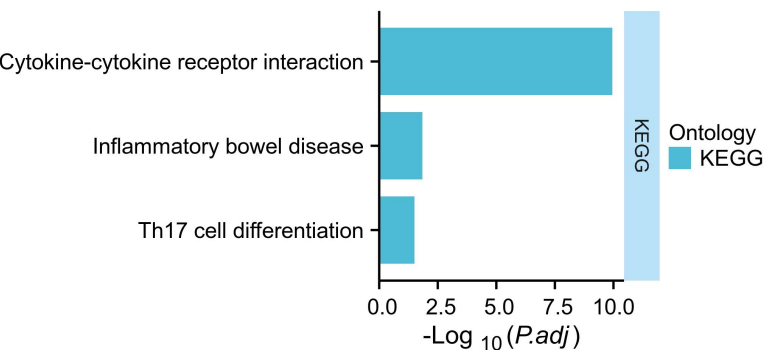**D**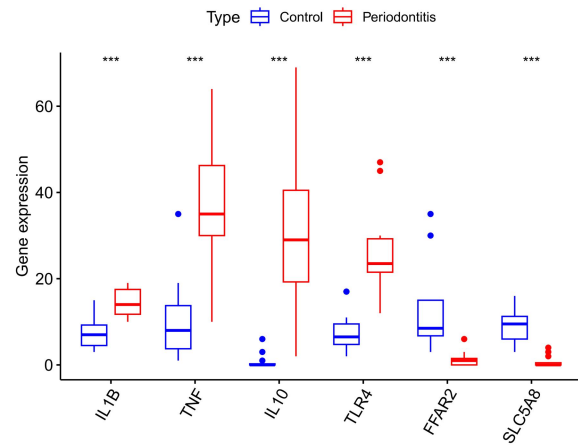

Supplement: Supplementary file 2 — Supporting Information 2 Figure S1: Quality assessment of RNA‐seq samples and key gene expression analysis in inflammatory and metabolic pathways. (A) PCA plot showing clear separation between periodontitis and healthy control samples along the PC1 and PC2 dimensions; (B) GO enrichment analysis highlighting significant immune‐ and inflammation‐related biological processes; (C) KEGG pathway enrichment analysis indicating the signaling pathways involving DEGs; (D) boxplots comparing the expression levels of key inflammation‐related cytokines (IL‐1β, TNF‐α, and IL‐10) and metabolic receptor genes (SLC5A8, GPR43, and TLR4) between groups. GCF samples were obtained from 12 patients with periodontitis and 12 healthy controls. ∗Indicates between‐group comparisons; ∗∗∗ p < 0.001. [file MI-2026-3542645-s002.pdf]

GPR43 (FFAR2) normalized expression

M1 macrophage proportion (CIBERSORT)

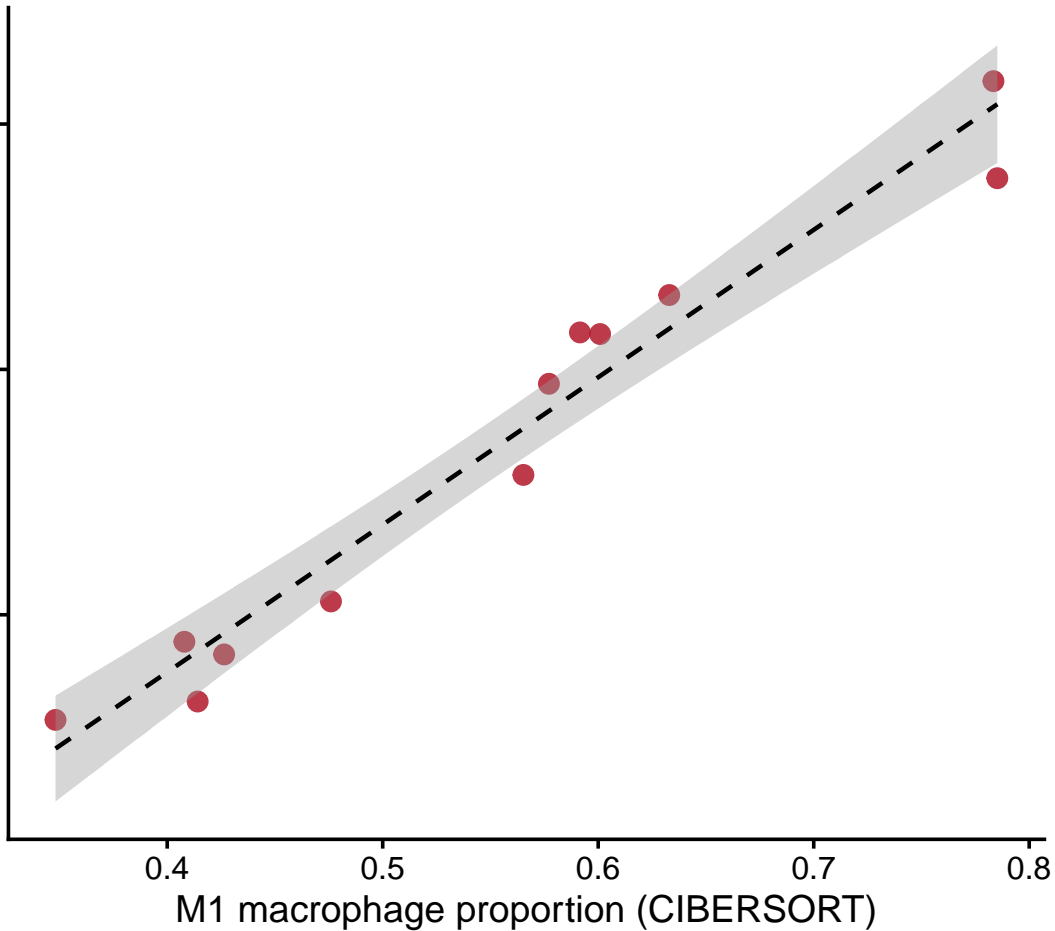

Supplement: Supplementary file 3 — Supporting Information 3 Figure S2: Correlation between GPR43 expression and M1 macrophage proportion in human periodontitis samples. Note: Based on bulk RNA sequencing data from human gingival tissues, the relationship between the expression level of the metabolite‐sensing receptor gene GPR43 (FFAR2) and immune cell deconvolution results was analyzed. The proportion of M1 macrophages was inferred using CIBERSORT. Correlation analysis was performed exclusively in periodontitis samples using Spearman’s rank correlation test. Although GPR43 expression exhibited an overall downregulation trend at the tissue level, its expression within periodontitis samples increased with higher proportions of M1 macrophages, suggesting that bulk transcriptomic results may be influenced by changes in cellular composition. [file MI-2026-3542645-s001.pdf]

**A**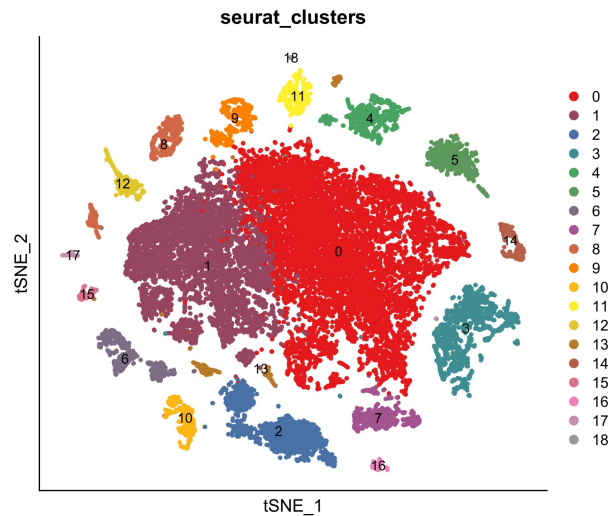**B**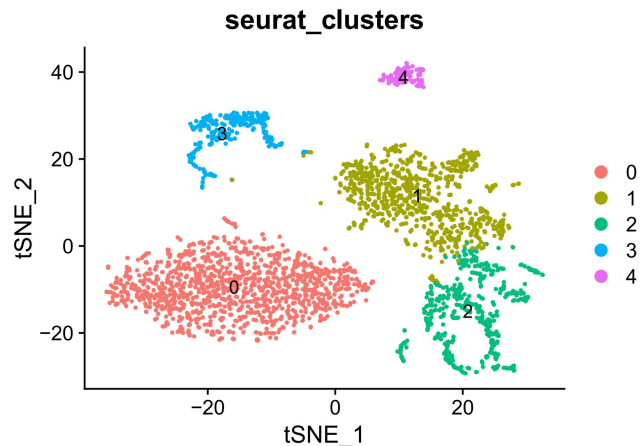**C**

GO/KEGG Enrichment of M1 Macrophages

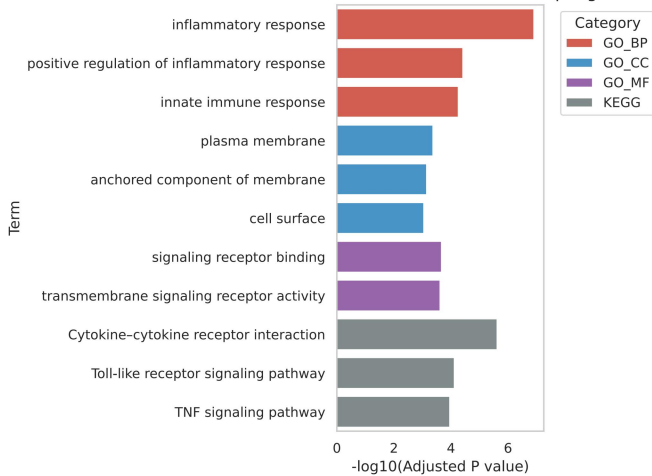**D**

GO/KEGG Enrichment of M2 Macrophages

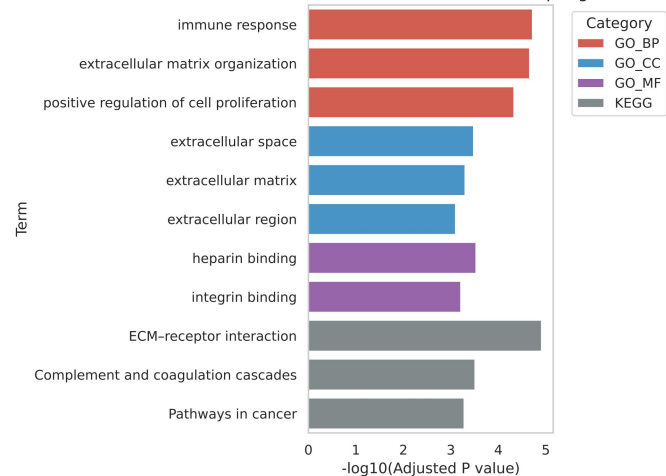

Supplement: Supplementary file 4 — Supporting Information 4 Figure S3: Clustering results and functional enrichment analysis of all cells and MC. (A) t‐SNE clustering map of all cells, identifying 19 clusters with distinct transcriptomic profiles; (B) secondary dimensionality reduction and clustering of MC, resulting in five major subclusters; (C) GO and KEGG pathway enrichment analysis of genes upregulated in M1 macrophages; (D) GO and KEGG pathway enrichment analysis of genes upregulated in M2 macrophages. [file MI-2026-3542645-s004.pdf]
